# Comparative chloroplast genome analysis of *Artemisia* (Asteraceae) in East Asia: insights into evolutionary divergence and phylogenomic implications

**DOI:** 10.1186/s12864-020-06812-7

**Published:** 2020-06-22

**Authors:** Goon-Bo Kim, Chae Eun Lim, Jin-Seok Kim, Kyeonghee Kim, Jeong Hoon Lee, Hee-Ju Yu, Jeong-Hwan Mun

**Affiliations:** 1 Department of Bioscience and Bioinformatics, Myongji University, Yongin, 17058 Korea; 2 National Institute of Biological Resources, Incheon, 22689 Korea; 3 Department of Herbal Crop Research, National Institute of Horticultural and Herbal Science, Chungbuk, 27709 Korea; 4 Department of Life Science, the Catholic University of Korea, Bucheon, 14662 Korea

**Keywords:** *Artemisia*, Asteraceae, Plastome, Evolution, *accD*, *ycf1*, Marker

## Abstract

**Background:**

*Artemisia* in East Asia includes a number of economically important taxa that are widely used for food, medicinal, and ornamental purposes. The identification of taxa, however, has been hampered by insufficient diagnostic morphological characteristics and frequent natural hybridization. Development of novel DNA markers or barcodes with sufficient resolution to resolve taxonomic issues of *Artemisia* in East Asia is significant challenge.

**Results:**

To establish a molecular basis for taxonomic identification and comparative phylogenomic analysis of *Artemisia*, we newly determined 19 chloroplast genome (plastome) sequences of 18 *Artemisia* taxa in East Asia, de novo-assembled and annotated the plastomes of two taxa using publicly available Illumina reads, and compared them with 11 *Artemisia* plastomes reported previously. The plastomes of *Artemisia* were 150,858–151,318 base pairs (bp) in length and harbored 87 protein-coding genes, 37 transfer RNAs, and 8 ribosomal RNA genes in conserved order and orientation. Evolutionary analyses of whole plastomes and 80 non-redundant protein-coding genes revealed that the noncoding *trnH-psbA* spacer was highly variable in size and nucleotide sequence both between and within taxa, whereas the coding sequences of *accD* and *ycf1* were under weak positive selection and relaxed selective constraints, respectively. Phylogenetic analysis of the whole plastomes based on maximum likelihood and Bayesian inference analyses yielded five groups of *Artemisia* plastomes clustered in the monophyletic subgenus *Dracunculus* and paraphyletic subgenus *Artemisia*, suggesting that the whole plastomes can be used as molecular markers to infer the chloroplast haplotypes of *Artemisia* taxa. Additionally, analysis of *accD* and *ycf1* hotspots enabled the development of novel markers potentially applicable across the family Asteraceae with high discriminatory power.

**Conclusions:**

The complete sequences of the *Artemisia* plastomes are sufficiently polymorphic to be used as super-barcodes for this genus. It will facilitate the development of new molecular markers and study of the phylogenomic relationships of *Artemisia* species in the family Asteraceae.

## Background

The genus *Artemisia* L. is the largest group in the tribe Anthemideae of the family Asteraceae, consisting of approximately 500 species [1, 2]. *Artemisia* species are widely distributed in the temperate regions of the Northern Hemisphere, including Europe, Asia, and North America, and a few species are reported from the Southern Hemisphere [3–5]. Many *Artemisia* taxa have been used as food, forage, ornamental, or soil stabilizers [6]. Moreover, several *Artemisia* species are used as traditional medicinal herbs for their high accumulation of essential oils and terpenoids with anti-malaria, anti-cancer, and anti-diabetes effects. For instance, artemisinin isolated from *A. annua* is widely used against malaria [7].

The center of origin and diversification of the genus *Artemisia* is Asia [8]. In East Asia, approximately 150 *Artemisia* species in two subgenera (subgenus *Artemisia* and subgenus *Dracunculus*) were described from East China, Korea, and Japan [9–11], many of which are used as supplements for medicinal or health purposes. For example, dried young leaves of different *Artemisia* species are collectively termed as Aeyeop (*A*. *argyi*, *A. montana*, and *A. princeps*), Haninjin (*A. gmelinii*), Cheongho (*A. annua* and *A*. *apiacea*), and Injinho (*A. capillaris*) in Korea [12]. To establish the taxonomic delimitation and phylogenetic relationships among the *Artemisia* taxa, a number of classical studies based mainly on the capitula type and floret fertility have been reported describing five subgeneric or sectional groups [*Artemisia*, *Absinthium* (Miller) Less, *Dracunculus* (Besser) Rydb., *Seriphidium* Besser ex Less., and *Tridentatae* (Rydb.) McArthur] [1, 5, 13]. However, taxonomic classification of *Artemisia* species has been controversial due to the insufficient diagnostic characters, highly variable morphological traits, potential natural hybridization among taxa, polyploidy, and nomenclatural legacy [1, 5, 8, 14–16]. Meanwhile, sequencing of nuclear and organelle genome regions, such as the external and internal transcribed spacer (ETS and ITS) of nuclear ribosomal DNA [8, 16, 17] and intergenic spacers between genes of chloroplast genome (plastome) [4, 18], has enabled molecular phylogenetic analyses of *Artemisia.* DNA markers widely applied to phylogenetic studies of *Artemisia* at the genus level include ITS, ITS2, *psbA*-*trnH*, *matK*, and *rbcL*. For example, the section *Tridentatae*, endemic to North America, was separated from the subgenus *Seriphidium* with strong support of ITS sequences [16, 19]. Recently, the subgenus *Pacifica*, including Hawaiian species, was recognized by nuclear ribosomal (ITS and ETS) and chloroplast (*trnL-F* and *psbA-trnH*) markers [20]. However, the resolution of these markers was insufficient to resolve taxonomic issues at the species level due to high sequence similarity of closely related taxa presumably caused by rapid radiation and hybridization [21–24]. Therefore, development of novel DNA markers or barcodes for investigation of *Artemisia* is an important challenge.

Chloroplasts are multifunctional plant-specific organelles that carry out photosynthesis and have roles in plant growth and development, such as in nitrogen metabolism, sulfate reduction, and synthesis of starch, amino acids, fatty acids, nucleic acids, chlorophyll, and carotenoids [25]. Chloroplasts of the plant kingdom arose from a single ancestral cyanobacterium [26]. In general, the plastomes of most plants are 120–160 kilobases (kb) in length and have a quadripartite structure comprising a large single copy (LSC), a small single copy (SSC), and two inverted repeat (IR) regions. The small and relatively constant size, conserved genome structure, and uniparental inheritance of the plastome make it an ideal genetic resource for phylogenetic analysis and molecular identification of higher plants (reviewed in [27]). Several variable regions of the plastome have been developed as DNA barcode marker systems to identify taxa. The chloroplast DNA barcode markers generated for plants include coding sequences within the plastome such as *matK*, *ndhF*, *rbcL*, *rpoB*, and *rpoC1* and the intergenic regions (IGRs) between *atpF*-*atpH*, *psbK*-*psbI*, and *trnH*-*psbA* [28, 29]. Of particular importance is a combination of *rbcL* and *matK*, which was recommended as a core barcode of land plants by the CBOL Plant Working group [28]. Additionally, *ycf1a* and *ycf1b* have been proposed as chloroplast barcodes due to their ease amplification by polymerase chain reaction (PCR) and abundant variations in land plants [30].

Recent advances in genome sequencing based on next generation sequencing (NGS) technologies and bioinformatics tools have increased the number of whole plastome sequences deposited in the public databases. This enables application of the plastome as a super-barcode for high-resolution phylogenetic analysis and species identification [31]. As of March 2020 (RefSeq Release 99), a total of 4718 chloroplast or plastid genomes of diverse species were deposited at the National Center for Biotechnology Information (NCBI) organelle genome database [32]. Among them, 11 plastomes of *Artemisia* species, *A. annua* L., *A. argyi* H. Lev. & Vaniot, *A. argyrophylla* Ledeb., *A. capillaris* Thunberg., *A. frigida* Willd., *A. fukudo* Makino, *A. gmelinii* Webb ex Stechmann, *A. montana* (Nakai) Pamp., and *A. princeps* Pamp. were included (Table 1). Comparative plastome analysis of these species identified mutational hotspots from intergenic spacer regions and showed that the genus *Artemisia* is a monophyletic genus and is a sister to the genus *Chrysanthemum* [40]. Additionally, the draft nuclear genome sequence of *A. annua* [2n = 2x = 18, 1.76 gigabases (Gb)/1C] covering 1.74 Gb was reported [41]. Although few chloroplast or nuclear genomes of *Artemisia* species are available, they are useful resources for studies of *Artemisia* and will enable the development of a novel *Artemisia* DNA marker system by comparative sequence analysis.

**Table 1:** Samples and assembly statistics of the *Artemisia* plastomes

Subgenus	Section	Scientific name	Nucleotide length (bp)				Number of genes			Reference or Voucher^a^	Genbank Accession
			Total	LSC	SSC	IR	Protein	tRNA	rRNA		
*Artemisia*	*Abrotanum*	*A. annua*	150,952	82,772	18,268	24,956	87	37	8	Zhang et al. 2017 (direct submission)	KY085890
		*A. annua*	150,955	82,776	18,267	24,956	87	37	8	Shen et al. 2017 [33]	MF623173
		*A. annua*	150,955	82,776	18,267	24,956	87	37	8	NIBRVP0000595661	MG951482
		*A. apiacea*	151,091	82,830	18,343	24,959	87	37	8	NIBRVP0000538751	MG951483
		*A. freyniana* f. *discolor*	151,275	82,965	18,344	24,983	87	37	8	NIBRVP0000538858	MG951487
		*A. fukudo*	151,011	82,751	18,348	24,956	87	37	8	Lee et al. 2016a [34]	KU360270
		*A. fukudo*	151,022	82,762	18,348	24,956	87	37	8	NIBRVP0000597993	MG951488
		*A. gmelinii*	151,247	82,988	18,341	24,959	87	37	8	NIBRVP0000592776	MG951489
		*A. gmelinii*	151,318	83,061	18,339	24,959	87	37	8	Lee et al. 2016b [35]	NC031399
	*Absinthium*	*A. frigida*	151,103	82,790	18,415	24,949	87	37	8	SRR8208356^b^	n.a.
		*A. frigida*	151,076	82,740	18,396	24,970	87	37	8	Liu et al. 2013 [36]	NC020607
		*A. nakaii*	151,020	82,760	18,348	24,956	87	37	8	NIBRVP0000598807	MG951494
		*A. sieversiana*	150,910	82,710	18,304	24,948	87	37	8	NIBRVP0000592824	MG951499
	*Artemisia*	*A. argyi*	151,176	82,915	18,347	24,957	87	37	8	NIBRVP0000592833	MG951484
		*A. argyi*	151,192	82,930	18,348	24,957	87	37	8	Kang et al. 2016 [37]	NC030785
		*A. argyrophylla*	151,189	82,927	18,348	24,957	87	37	8	Kim et al. 2017 (direct submission)	MF034022
		*A. feddei*	151,112	82,878	18,322	24,956	87	37	8	NIBRVP0000592740	MG951486
		*A. keiskeana*	150,858	82,622	18,344	24,946	87	37	8	NIBRVP0000592791	MG951492
		*A. montana*	151,150	82,891	18,345	24,957	87	37	8	NIBRVP0000627850	MG951493
		*A. montana*	151,130	82,873	18,343	24,957	87	37	8	Choi and Park, 2014 (direct submission)	NC025910
		*A. princeps*	151,193	82,932	18,347	24,957	87	37	8	NIBRVP0000592810	MG951495
		*A. rubripes*	151,133	82,874	18,345	24,957	87	37	8	NIBRVP0000592774	MG951496
		*A. selengensis*	151,255	82,942	18,389	24,962	87	37	8	NIBRVP0000538775	MG951497
		*A. selengensis*	151,261	82,948	18,389	24,962	87	37	8	NIBRVP0000595650	MG951498
		*A. selengensis*	151,215	82,920	18,371	24,962	87	37	8	Meng et al. 2019 [38]	MH042532
		*A. stolonifera*	151,144	82,878	18,350	24,958	87	37	8	NIBRVP0000592785	MG951500
*Dracunculus*	*Dracunculus*	*A. capillaris*	151,020	82,790	18,306	24,962	87	37	8	Kim et al. 2017 (direct submission)	KY073391
		*A. capillaris*	151,020	82,790	18,306	24,962	87	37	8	NIBRVP0000592735	MG951485
		*A. capillaris*	151,056	82,821	18,313	24,961	87	37	8	Lee et al. 2016b [35]	NC031400
		*A. dracunculs*	151,042	82,811	18,317	24,957	87	37	8	SRR8208350^c^	n.a.
	*Latilobus*	*A. hallaisanensis*	151,015	82,823	18,290	24,951	87	37	8	NIBRVP0000538771	MG951490
		*A. japonica*	151,080	82,844	18,314	24,961	87	37	8	NIBRVP0000592828	MG951491

^a^Vouchers were deposited at the National Institute of Biological Resources (Incheon, Korea)

^b, c^Raw sequence reads were downloaded from NCBI SRA database [39] and de novo assembled in this study

We aimed to identify variable regions in the plastomes of the *Artemisia* taxa in East Asia to establish a molecular basis for the development of novel DNA barcode markers that can be widely applicable across the genus *Artemisia* as well as the family Asteraceae. We newly sequenced and assembled 19 plastomes of 18 taxa from two subgenera of *Artemisia*. Additionally, we de novo*-*assembled and annotated two plastomes using publicly available NGS reads. Combined with 11 previously reported *Artemisia* plastomes, we performed a comparative analysis of 32 *Artemisia* plastomes and identified highly variable regions in the *Artemisia* plastomes. Our results provide a robust genomic framework for taxonomic and phylogenomic characterization of *Artemisia* species in East Asia and the development of DNA markers that allow identification of individual taxa in a cost-effective manner.

## Results

### Structure and features of the *Artemisia* plastomes

A total of 32 complete plastomes from 21 *Artemisia* taxa were analyzed (Table 1). These taxa belong to the sections *Abrotanum*, *Absinthium*, and *Artemisia* of the subgenus *Artemisia* and the sections *Dracunculus* and *Latilobus* of the subgenus *Dracunculus* [5, 6, 11]. Among them, 19 plastomes from 18 taxa were newly sequenced and assembled in this study. To assemble the plastomes, we generated approximately 35.2 million Illumina MiSeq PE reads (10.6 Gb) on average per sample (Additional file 2: Table S1). De novo assembly of the Illumina reads using *rbcL* and *rpoC2* of *A. argyi* (GenBank accession NC030785) as seed sequences resulted in the construction of a circular DNA sequence map for each sample. Additionally, the Sequence Read Archive (SRA) reads of *A. dracunculus* (SRR8208350) and *A. frigida* (SRR8208356) deposited in NCBI were de novo assembled into circular plastomes. The 21 de novo*-*assembled plastomes were verified by mapping of sequence reads affording 666-fold average coverage (296-fold to 1187-fold coverage). The remaining 11 plastomes from 9 *Artemisia* species were downloaded from NCBI. The structural orientation of the LSC, SSC, and IR regions of each assembly was analyzed by comparison with previously reported *Artemisia* plastomes. As a result, we obtained at least two independent plastome assemblies for each of eight species (*A. annua*, *A. argyi*, *A. capillaris*, *A. frigida*, *A. fukudo*, *A. gmelinii*, *A. montana*, and *A. selengensis*) and a single plastome for each of 13 taxa (*A. apiacea*, *A. argyrophylla*, *A. dracunculus*, *A. feddei*, *A. freyniana f. discolor*, *A. hallaisanensis*, *A. japonica*, *A. keiskeana*, *A. nakaii*, *A. princeps*, *A. rubripes*, *A. sieversiana*, and *A. stolonifera*).

**Fig. 1:**
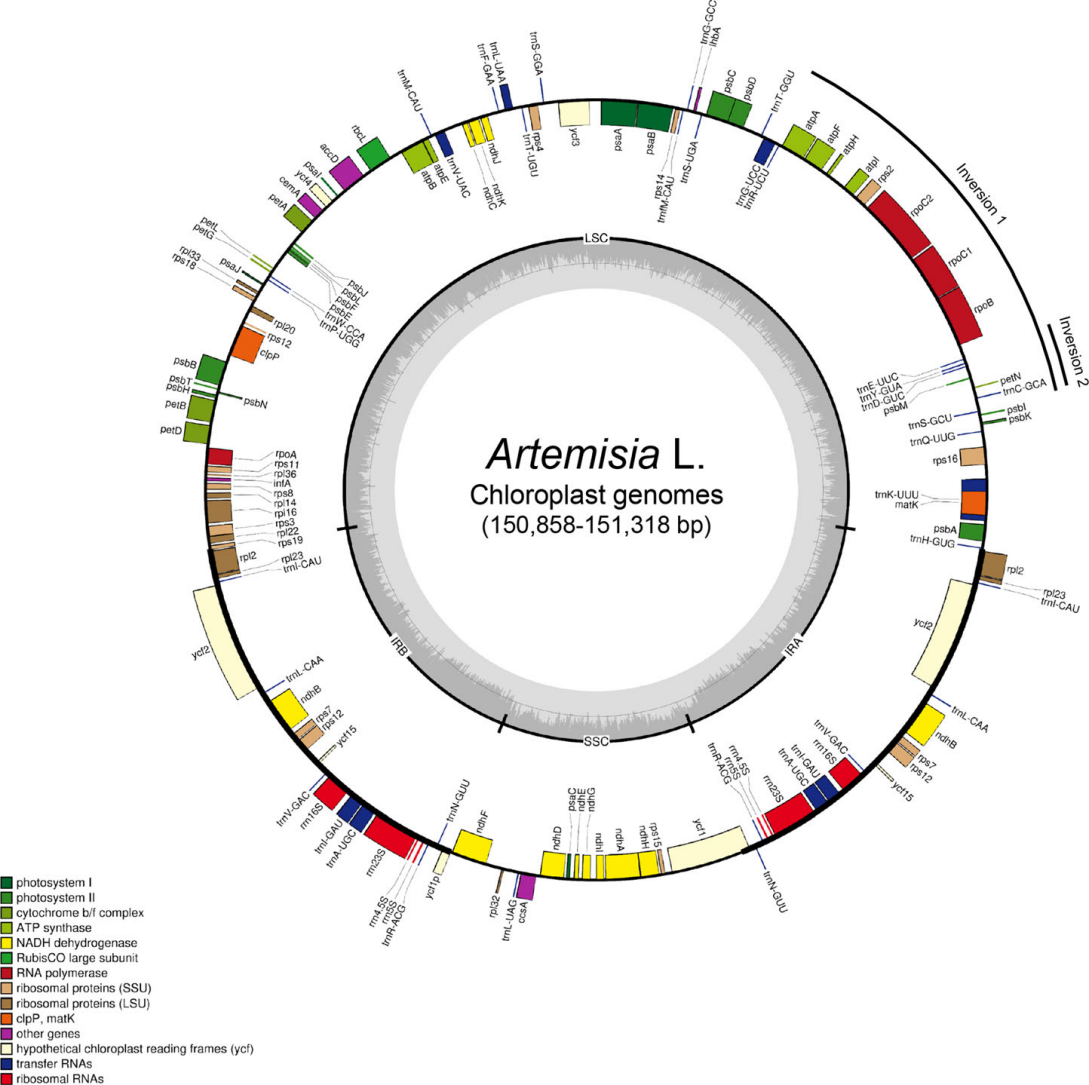
A circular gene map of the *Artemisia* plastomes. Circle 1 (from inside) indicates the GC content. The colored bars on circle 2 indicate protein-coding genes, tRNA genes, and rRNA genes. Genes are placed on the inside or outside of circle 2 according to their orientations. Functional categories of genes are presented in the left margin. IR, inverted repeat region; LSC, large single copy region; SSC, small single copy region

The de novo*-*assembled *Artemisia* plastomes were 150,858 bp (*A. keiskeana*) to 151,318 bp (*A. freyniana f. discolor*) in length with a 37.4–37.5% GC content, similar to previously reported *Artemisia* plastomes. They had a typical quadripartite structure consisting of 82,622–82,988 bp of LSC, 24,946–24,983 bp of SSC, and a pair of IRs, each of which was 18,267–18,389 bp (Fig. 1). Comparing with the plastome of *Nicotiana tabacum* (GenBank accession NC001879), all the *Artemisia* plastomes had two inversions (approximately 22 kb and 3.3 kb in length) in the LSC region that have been reported to be shared by all clades of the Asteraceae family (Fig. 1) [42]. Gene annotation showed that the *Artemisia* plastomes contained 87 protein-coding genes, 37 transfer RNAs (tRNAs), and 8 ribosomal RNA (rRNA) genes in conserved order and orientation (Table 1). Comparison of plastome sequences from the same species, except *A. capillaris* (GenBank accession KY073391 and MG951485), identified three bp (*A. annua*) to 71 bp (*A. frigida*) length differences that are randomly distributed both in genic and non-genic regions. In every *Artemisia* plastome, the junctions between IRs and LSC and SSC were flanked by *rps19* and *ycf1*, respectively (Additional file 1: Fig. S1). The IR border structure was conserved in *Artemisia*, except *A. selengensis* in which three independent plastomes have seven bp expansion in *rps19* at the LSC/IR and SSC/IR junctions. In addition, unlike the reports of Meng et al. [38] and Shen et al. [33], *ψrps19* was located at the IRb/LSC junction in all *Artemisia* plastomes. Seven protein-coding genes (*ndhB*, *rpl2*, *rpl23*, *rps7*, *rps12*, *ycf2*, and *ycf15*), four rRNA genes, and seven tRNA genes were duplicated in the two IRs. Moreover, 12 protein-coding genes and six tRNA genes had one or two introns (Additional file 2: Table S2). Of the total plastomes, protein-coding genes comprised 52.3% whereas rRNA and tRNA genes accounted for 6.0 and 1.9%, respectively. We found several annotation errors in the previously reported sequences. For example, two pseudogenes, *ψycf1* and *ψrps19*, were newly identified in all of the plastomes and *psbG* in *A. annua* (GenBank accession MF623173) was an erroneous annotation.

### Identification of polymorphisms in the *Artemisia* plastomes

A sequence comparison of 32 *Artemisia* whole plastomes generated multiple aligned sequences of 153,229 bp in length. The alignment exhibited high pairwise sequence identities between plastomes of the same section, ranging from 99.2% (section *Absinthium*) to 99.8% (section *Dracunculus*) in whole plastomes and from 99.7% (section *Absinthium*) to 99.9% (section *Dracunculus*) in the protein-coding genes. Interestingly, the protein-coding genes of *A. argyrophylla* (GenBank accession MF034022) in section *Artemisia* and *A. nakaii* (GenBank accession MG951494) in section *Absinthium* showed 100% identity with those of *A. argyi* (GenBank accessions MG951484 and NC030785) in section *Artemisia* and *A. fukudo* (GenBank accessions KU360270 and MG951488) in section *Abrotanum*, respectively (Additional file 2: Table S3).

**Fig. 2:**
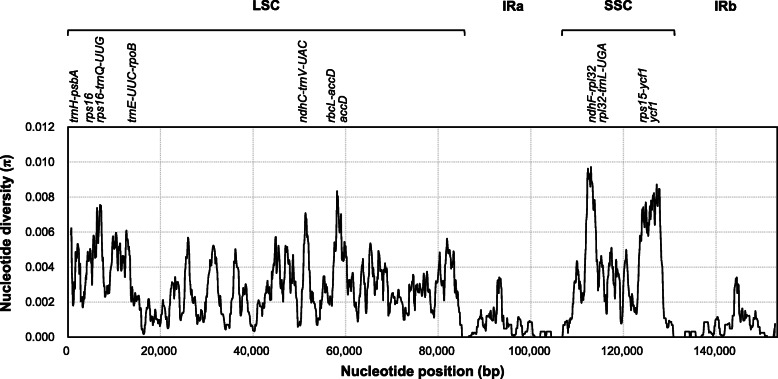
Sliding window test of nucleotide diversity (π) in the multiple alignments of the 32 *Artemisia* plastomes. Peak regions with a π value of > 0.006 were labeled with loci tags of genic or intergenic region names. π values were calculated in 1 kb sliding windows with 100 bp steps. LSC, large single copy region; IRa, inverted repeat region a; SSC, small single copy region; IRb, inverted repeat region b

A total of 2172 variable sites comprising 1062 singleton variable sites and 1110 parsimony informative (PI) sites (0.72%) were identified across the whole plastome alignment (Table 2). The overall nucleotide diversity (π) was 0.0024; however, each structural region of plastome showed different nucleotide diversities and PI sites; these were highest in SSC (π = 0.0047 and PI = 1.37%) and lowest in IR (π = 0.0006 and PI = 0.19%) regions. Based on DNA polymorphisms, the *Artemisia* plastomes could be divided into 30 chloroplast haplotypes along with 30 LSC, 26 SSC, and 23 IR haplotypes. Across the *Artemisia* plastomes, highly diverged regions were identified by calculating π values within 1 kb sliding windows with 100 bp steps (Fig. 2). In total, 11 peaks with π values higher than 0.006 were identified from the plastome. These regions included *trnH-psbA*, *rps16*, *rps16-trnQ-UUG*, *trnE-UUC-rpoB*, *ndhC-trnV-UAC*, *rbcL-accD*, and *accD* in LSC and *ndhF-rpl32*, *rpl32-trnL-UAG*, *rps15-ycf1*, and *ycf1* in SSC regions (Additional file 2: Table S4 and S5). Sequence analysis of three highly diverged protein-coding genes (*accD*, *ycf1*, and *rps16*) revealed high polymorphisms (π > 0.006) in the coding sequences of *accD* and *ycf1* and in the intron of *rps16*.

**Table 2:** DNA polymorphisms identified in the 32 *Artemisia* plastomes

Structural region	Alignment length (bp)	Number of variable sites			Nucleotide polymorphism		
		Polymorphic	Singleton	PI^a^	PI sites (%)	π^b^	H^c^
Whole DNA	153,229	2172	1062	1110	0.72	0.0024	30
LSC	84,443	1501	742	759	0.90	0.0029	30
SSC	18,737	523	266	257	1.37	0.0047	26
IR^d^	50,049	148	54	94	0.19	0.0006	23

^a^Parsimony informative; ^b^nucleotide diversity; ^c^number of haplotypes

^d^Alignments of two IR regions were combined and the 7 bp expansion in *A. selengensis* was included

For 80 non-redundant protein-coding genes, a total of 68,062 bp sequences were multiply aligned. The overall nucleotide diversity of protein-coding genes (π = 0.0015) was approximately 1.6-fold lower than that of whole plastome (π = 0.0024). Notably, 17 genes had a higher π than the overall π value and showed an average 99.5% pairwise sequence similarity of coding sequences (Table 3). The PI sites of these genes comprised 39.2% (144 of 367 sites) of the total PI sites in all protein-coding genes. Of particular interest, *accD*, encoding the beta-carboxyl transferase subunit of acetyl-CoA carboxylase, and *ycf1*, encoding Tic214 of the TIC complex, showed lower sequence identity, higher nucleotide diversity, and a larger number of PI sites than the other genes, indicating a high level of sequence divergence. Additionally, *ndhF* and *rpoC2* had more than ten PI sites; however, their π values were lower than 0.003. Therefore, two protein-coding genes, *accD* and *ycf1*, were identified as nucleotide diversity hotspots of the *Artemisia* chloroplast protein-coding genes, and have potential as candidate regions for the development of universal barcode markers.

**Table 3:** Evolutionary characteristics of 17 highly diverged protein-coding genes in the *Artemisia* plastome

Gene^a^	Length of alignment (bp)	Avg. pairwise similarity (%)^b^	Identical sites (%)	π	H	Total variable sites	Singleton sites	PI sites	Ka/Ks^c^
*ycf1*	5076	98.96	94.2	0.0065	24	44	21	23	0.6674
*accD*	1572	98.7	92.8	0.0057	19	42	12	30	1.0568
*infA*	231	99.63	97.9	0.0037	7	5	2	3	0.0097
*ndhE*	303	99.63	98.4	0.0036	6	5	2	3	0.0295
*rps8*	402	99.67	98.5	0.0033	7	6	1	5	0.3830
*ndhF*	2223	99.7	98.5	0.0030	18	32	9	23	0.1783
*psaC*	243	99.71	98.4	0.0029	5	4	2	2	0
*petD*	480	99.71	98.3	0.0029	8	8	4	4	0.0112
*rpl22*	471	99.66	97.3	0.0027	9	7	3	4	0.1161
*psbT*	99	99.76	97.1	0.0025	4	3	3	0	0
*rpl16*	405	99.73	98.8	0.0022	6	5	1	4	0
*rpl36*	111	99.8	98.2	0.0020	2	2	0	2	0
*matK*	1515	99.52	97.6	0.0019	16	20	11	9	0.2803
*rps3*	654	99.69	98.3	0.0018	12	11	4	7	0.1808
*psbK*	177	99.67	98.9	0.0017	3	2	1	1	0.1924
*rpoC2*	4137	99.8	98.5	0.0017	22	56	36	20	0.3194
*petB*	645	99.78	98.8	0.0016	9	8	4	4	0
Overall	68,214	99.50	98.2	0.0015	28	769	402	367	0.1774

^a^Genes with > 0.2% average pairwise dissimilarity and > 0.0015 π values were selected

^b^Coding sequences were aligned using MUSCLE and translational alignment in Geneious Prime

^c^Ka/Ks values (ω) were calculated according to Yang and Nielsen (2000) [43] using the yn00 program in the PAML 4 package

### Variation and evolutionary selection of protein-coding genes

No gene loss was detected from the 32 *Artemisia* plastomes; however, single nucleotide insertion or deletion (InDel) mutations resulting in a premature stop codon were found in *rpoA* of *A. montana* (GenBank accession MG951493) and *ycf1* of *A. selengensis* (GenBank accession MH042532), respectively. The frameshift caused by single nucleotide InDels generated truncated coding sequences, 816 bp instead of 1009 bp for *rpoA* of *A. montana* and 1290 bp rather than 5033 bp for *ycf1* of *A. selengensis*. In *A. sieversiana* (GenBank accession MG951499), one SNP in *ndhI* induces an in-frame premature stop codon, resulting in loss of eight codons at the 3′-end of the open reading frame.

**Fig. 3:**
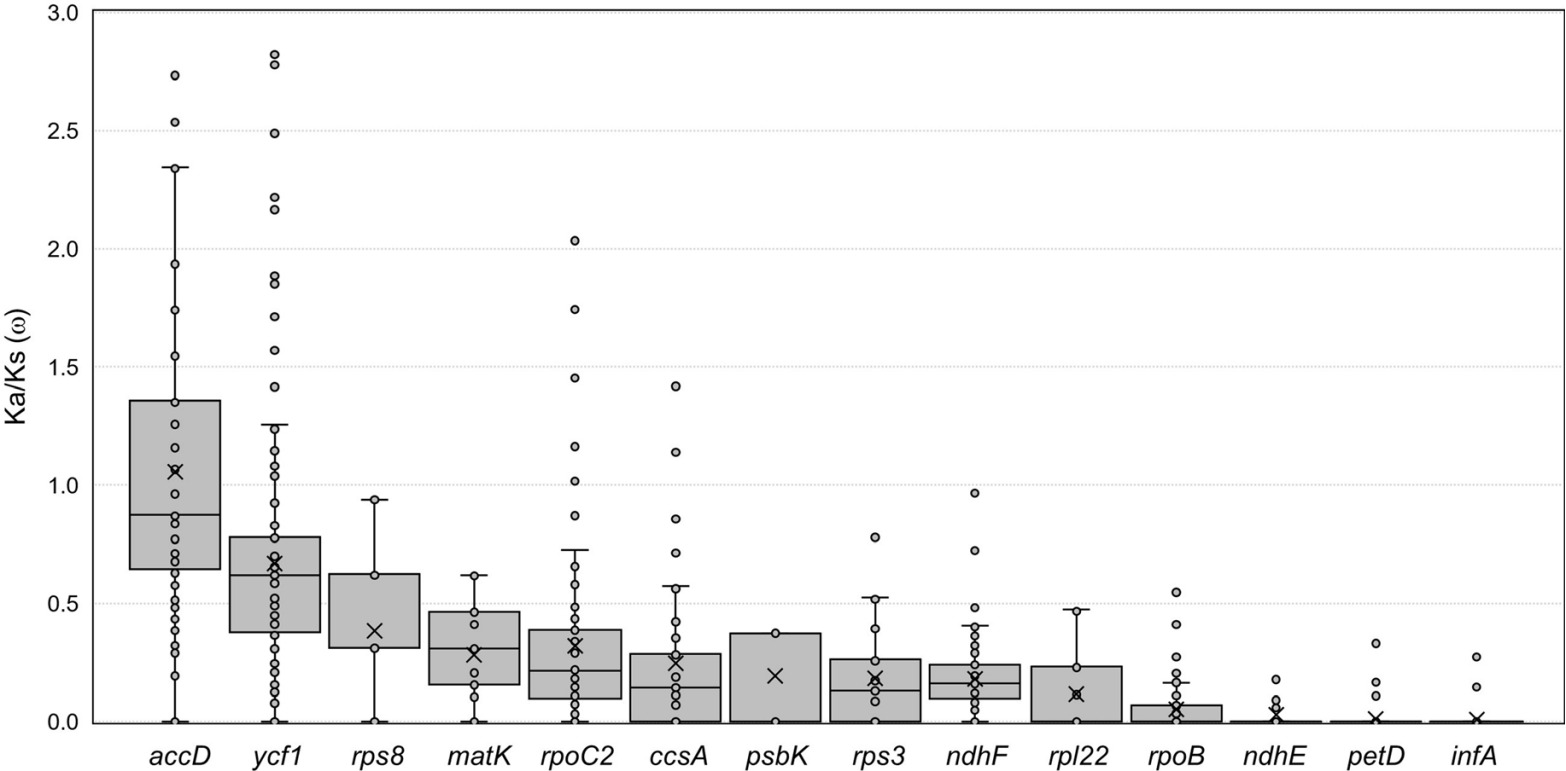
Box-and-whisker plots of the Ka/Ks (ω) values of highly diverged protein-coding genes in the *Artemisia* plastomes. The pairwise ω values of 14 protein-coding genes in the 32 *Artemisia* plastomes were calculated and plotted. Box plots show the median (central line), mean (× symbol), first and third quartiles (top and bottom bars), and outliers

Synonymous (Ks) and non-synonymous substitution rates (Ka) are useful for inferring the evolutionary tendency of genes. To evaluate differences in the selection and evolution of protein-coding genes in the *Artemisia* plastomes, the nucleotide substitution rates and average Ka/Ks ratio (ω) of 17 highly divergent genes were calculated. As shown in Table 3 and Fig. 3, 15 genes exhibited ω values less than 0.5, suggesting the action of high selective constraints or purifying selection. In contrast, the ω for *ycf1* and *accD* was 0.67 and 1.06, respectively, suggesting that these genes are under relaxed selective constraints and weak positive selection, respectively. These results are consistent with reports that most genes in the *Artemisia* plastome evolve under negative selection; however, *accD* is under positive selection [38, 44]. The likelihood ratio test of the site-specific model in CodeML program validated the evolutionary selection patterns of *accD* and *ycf1*. The Bayes empirical Bayes (BEB) identified 8 amino acid sites from *accD* and *ycf1*, respectively, that were positively selected under posterior probability > 0.95 (Additional file 2: Table S6). In *accD*, six out of the eight positively selected amino acid substitutions were located near *A. selengensis*-specific insertions consisting of 6 codon sequences repeating three or four times (Additional file 1: Fig. S2). This region is a polymorphic hotspot of *accD*. In contrast, the positively selected amino acid substitutions in *ycf1* were widely distributed across the coding sequences.

### Repetitive sequences in the *Artemisia* plastomes

**Fig. 4:**
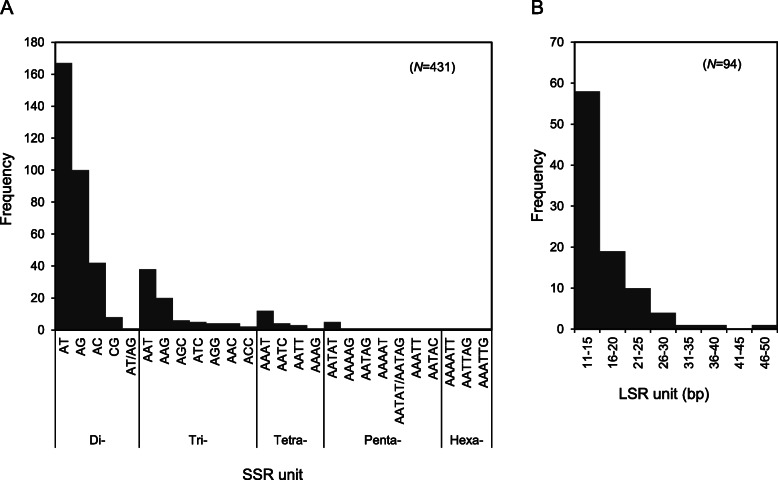
Frequency of repetitive sequences in the *Artemisia* plastomes. **a** Frequency of SSRs with di- to hexa-nucleotide motifs. **b** Frequency of LSRs with 11–50 nucleotide repeat units

Repeated DNA sequences in the plastome play a role in genome rearrangement and are useful for phylogenetic studies. We investigated simple sequence repeats (SSRs) and long sequence repeats (LSRs) in the multiple alignment of the 32 *Artemisia* whole plastomes (Table 4 and Fig. 4). A total of 431 SSR loci of short (2–6 bp) nucleotide motifs were discovered. Approximately 39.0% (168 of 431 loci) of SSR loci were in the protein-coding sequences. Moreover, only 27 SSR loci (6.2%) were polymorphic across the 32 plastomes; all were located in LSC and SSC, but none in IR, regions. In the *Artemisia* plastomes, di- and trinucleotide repeats were the most frequent, accounting for 72.2 and 18.4% of the total SSRs, respectively (Fig. 4a).

**Table 4:** Simple sequence repeats and long sequence repeats identified in the 32 *Artemisia* plastomes

Repeats		SSR				LSR			
Structural region		LSC	SSC	IR	Total	LSC	SSC	IR	Total
Numbers	Overall	296	60	75	431	62	22	10	94
	Polymorphic	22	5	0	27	37	12	3	52
	Monomorphic	274	55	75	404	25	10	7	42
Genic region	CDS	109	25	34	168	10	7	2	19
	Intron	39	5	10	54	3	2	0	5
	rRNA/tRNA	8	0	9	17	0	0	0	0
Intergenic region		140	30	22	192	49	13	8	70

A total of 94 LSRs were identified (Fig. 4b). Unlikely SSRs, more than half of them (55.3%) were polymorphic (Table 4). Among the polymorphic LSRs, those repeated twice (38 of 49) were most abundant. Palindromic and hairpin repeats were also found. LSRs were 2.3-fold as frequent in IGRs compared to genic regions. In each structural region, SSC region had the highest average density of LSRs per kilobase (1.4/kb), followed by LSC (1.2/kb) and IR (0.8/kb) regions. Most repeat units were less than 15 bp in length, which accounted for 81% of the total LSRs (Additional file 2: Table S7).

### Phylogenetic analysis and delimitation of the *Artemisia* plastomes

**Fig. 5:**
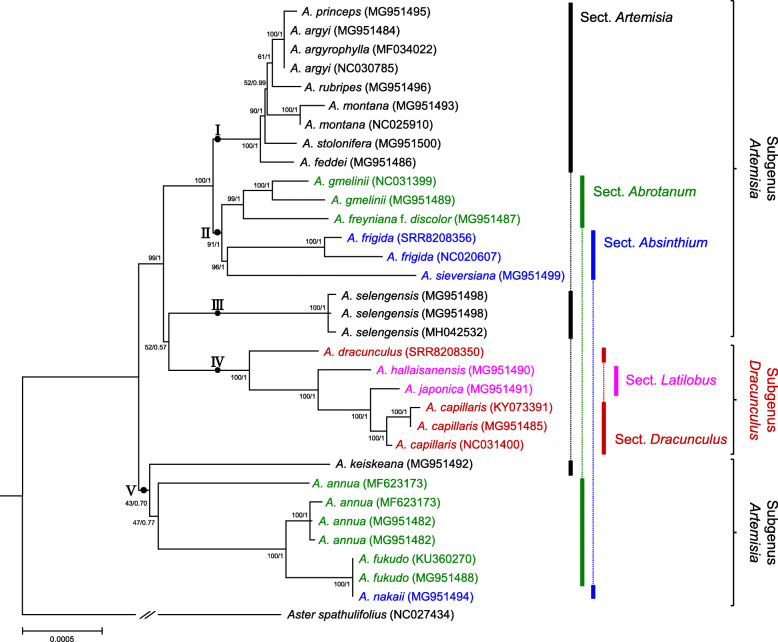
Phylogenetic tree of *Artemisia* taxa based on 80 non-redundant protein-coding genes of the plastome. BI tree topology is shown and BI posterior probability/ML bootstrap values are indicated on the nodes. Branch lengths were estimated by BI analysis. Colored lines and braces at the right of the tree indicate section and subgenus names of *Artemisia*

Using 80 protein-coding genes and the complete plastome sequences, we performed phylogenetic analyses of the 32 *Artemisia* plastomes. The topology of the maximum likelihood (ML) and Bayesian inference (BI) trees based on protein-coding genes was nearly identical (Fig. 5 and Additional file 1: Fig. S3). Additionally, no significant differences between the trees in protein-coding genes and whole plastomes were found, except clustering of *A. sieversiana* (MG951499). The analyses discriminated 19 of 21 (90.5%) analyzed plastomes and divided the *Artemisia* plastomes into five clades of two subgenera. The subgenus level classification of *Artemisia* based on plastomes was in agreement to the previous studies inferred from different types of DNA markers [4, 8, 16, 18, 19, 45]. The plastomes of the subgenus *Dracunculus* clustered together in monophyletic clade VI whereas those of the subgenus *Artemisia* were clustered into four paraphyletic clades (clades I − III and V). In contrast, plastomes in the same section were divided into different clades, showing that the previous section level classifications of *Artemisia* species [5, 6, 11, 17] were weakly supported by the plastome trees. All plastomes from the same species clustered together. Moreover, most plastomes of different taxa were distinct, except *A. argyi*, *A. argyrophylla*, and *A. princeps*, which clustered together in clade I with a near-zero branch length. These three species showed 99.97–99.99% sequence similarity to the whole plastome (Additional file 2: Table S3). Similarly, *A. nakaii* clustered with *A. fukudo* as expected from the plastome sequence similarity (99.99%). This finding supports the hypothesis that a number of *A. nakaii* accessions are likely to be interspecific hybrid taxa and its maternal origin might be *A. fukudo* [46].

### Molecular markers for *Artemisia* and the Asteraceae species

A comparative sequence analysis revealed that *accD* and *ycf1* are highly polymorphic in the *Artemisia* plastomes; therefore, these genes have potential for the development of *Artemisia* molecular markers. For *accD*, the 928 bp un-gapped alignment of the 1 kb region flanking the polymorphic hotspot was investigated as a molecular marker. For *ycf1*, we evaluated the potential utility of the Asteraceae *ycf1* locus by reconstructing a phylogenetic tree based on the coding sequences of 211 *ycf1* genes from Asteraceae whole plastomes in the NCBI nucleotide database. A ML tree constructed using the *ycf1* genes showed that the Asteraceae species were divided into nine groups; in agreement with the tribe level taxonomic classification of the Asteraceae (Additional file 1: Fig. S4) [47–49]. The topology of a ML tree based on 212 Asteraceae *accD* genes was similar (Additional file 1: Fig. S5). However, the resolution was insufficient for species delimitation. BLAST search of *accD* and *ycf1* against the Asteraceae plastomes revealed that these genes have higher tribe resolution (83.4% for *accD* and 99.1% for *ycf1*) than species resolution (42.4% for *accD* and 62.2 for *ycf1*). (Additional file 1: Fig. S6).

To develop *Artemisia* molecular markers with increased resolution of phylogeny reconstruction, we tested the combination of *accD* and *ycf1b*, a candidate plastid barcode for land plants developed based on the *ycf1* hotspot [30]. The *accD* marker is 928 bp in length (*accD*-1 k) and discriminated 19 *Artemisia* haplotypes (nucleotide diversity, π = 0.0077). Similarly, *ycf1b* is 847 bp in length and discriminated 18 haplotypes (π = 0.0080). The combination of *accD* and *ycf1b* (*accD*-1 k + *ycf1b*) increased the discriminatory power of the marker; 21 haplotypes were distinguished (π = 0.0080) (Table 5). The topology of the ML tree based on *accD*-1 k + *ycf1b* was similar to that of whole plastomes (Additional file 1: Fig. S7).

**Table 5:** Nucleotide diversity and discriminatory power of the *Artemisia* chloroplast markers

Loci	*trnH*-*psbA*	*accD*-hotspot	*ycf1b*	*accD*-1 k	*ycf1b* + *accD*-1 k
Length (bp)	357	276	847	928	1775
Number of sequence	32	32	32	32	32
Number of haplotype	18	11	18	19	21
Haplotype diversity	0.952	0.985	0.951	0.962	0.970
π	0.0137	0.0219	0.0080	0.0077	0.0080
Polymorphic site	25	23	38	47	52
PI site	15	9	21	36	33
Singleton site	10	1	17	11	19

To optimize the *accD*-1 k + *ycf1b* marker for the family Asteraceae, 212 *accD* and 211 *ycf1* genes of Asteraceae species retrieved from NCBI were analyzed. We compared the primer binding sites using multiple aligned sequences of each gene under a 95% threshold of consensus. The Asteraceae *ycf1* sequences at the 3′-end of the ycf1bF primer binding site [30] had five to six nucleotide mismatches. In contrast, the primer binding sites of ycf1bR as well as of the forward and reverse primers of *accD*-1 k were mostly conserved. Considering the consensus sequences, we designed and optimized primer pairs of the *accD*-1 k + *ycf1b* marker for Asteraceae species (Table 6). In silico estimation of PCR amplification under two stringency criteria predicted a 96–100% PCR success rate for the ycf1b-Asteraceae-F/R primer pair in the Asteraceae family (Additional file 2: Table S8) compared to 0% for the ycf1bF/R primer pair [30]. The accD-Asteraceae-F/R primer pair was predicted to have a 100% PCR success rate under both stringency conditions.

**Table 6:** Optimized *accD* and *ycf1* barcode primers for the family Asteraceae

Barcode	Primer		Sequence (5′ to 3′)
*accD*-1 k	Forward	Consensus at 95% threshold^a^	CGATGTTATTTAAGAMGGAGTTCG
		accD-Asteraceae-F^b^	CGATGTTATTTAAGAAGGAGTTCG
	Reverse	Consensus at 95% threshold^a^	CAAACGGGTAATTTTCTCCCC
		accD-Asteraceae-R^b^	CAAACGGGTAATTTTCTCCCC
	Expected amplicon size (bp)		928–1079
*ycf1b*	Forward	Consensus at 95% threshold^d^	RMTCGACGAAAATCYGGTTCTTCYAAAT
		ycf1b-Asteraceae-F^c^	GCTCGACGAAAATCCGGTTCTTCCAAAT
		ycf1bF^e^	TCTCGACGAAAATCAGATTGTTGTGAAT
	Reverse	Consensus at 95% threshold^d^	WTACATGYSAAAGTGATGGAAAA
		ycf1b-Asteraceae-R^c^	ATACATGCCAAAGTGATGGAAAA
		ycf1bR^e^	ATACATGTCAAAGTGATGGAAAA
	Expected amplicon size (bp)		874

^a^Consensus at 95% threshold across 212 *accD* sequences from the family Asteraceae

^b, c^Optimized primers for the family Asteraceae

^d^Consensus at 95% threshold across 211 *ycf1* sequences from the family Asteraceae

^e^Universal barcode primer set designed for angiosperm [30]

## Discussion

The accurate identification of plant species is important not only for taxonomy but also in agriculture and pharmaceuticals. The genus *Artemisia* includes a number of taxa of medicinal and economic value; however, classification and delimitation of individual species are challenging due to the insufficient morphological characters, frequent natural hybridization, and the presence of diverse intermediates [1, 5, 14–16]. Molecular marker techniques based on robust genomic tools could resolve issues in the species delimitation and phylogenetic relationships of *Artemisia*. As a universal barcode for plants, ITS1, ITS2, and ETS were developed into a sequence-characterized amplified region [50, 51] or high-resolution melt markers [52] and applied to *Artemisia*. However, insufficient sequence polymorphism of the markers and the frequent polyploidy of *Artemisia* hindered their use for species identification. In the previous comparative plastome analysis of the 11 *Artemisia* species, intergenic spacer regions, including *ccsA-ndhD*, *trnH-psbA*, *ndhG-ndhI*, *rps18-rpl20*, and *rps15-ycf1*, were identified as mutational hotspots. However, these loci have not been analyzed in the wide range of taxa of the Asteraceae family [40]. Additionally, known core chloroplast barcodes, such as *rbcL*, *matK*, *rpoB* and *rpoC1*, had low discriminatory power for classification of *Artemisia* taxa [21–23]. Therefore, discovery of novel barcodes with high discriminatory power or use of the whole plastome as a super-barcode is important. In this study, we investigated 32 whole plastomes from 21 *Artemisia* taxa native to East Asia, the largest number of *Artemisia* plastomes analyzed to date, to establish a basis for phylogenomics and DNA barcode development.

The *Artemisia* plastomes showed structural characteristics and genetic properties typical of the angiosperm plastome. The plastomes of *Artemisia* taxa are approximately 151 kb in length on average and organized into quadripartite regions with no structural variation among taxa. Each genome contains the same number of genes (87 protein-coding, 37 tRNA, and 8 rRNA genes) with a similar GC content and conserved intron positions. Such highly similar plastomes of *Artemisia* taxa enable multiple alignment of protein-coding genes and whole plastomes to identify regions with high sequence divergence. Interestingly, singly nucleotide InDel mutations resulting in frame-shift or premature stop codon were identified in *rpoA* of *A. montana* and *ycf1* of *A. selengensis*. In several species of Asteraceae, similar single nucleotide InDels were identified at the SSC/IR junction. For example, both *Chrysanthemum indicum* (GenBank accession NC020320) and *C. boreal* (GenBank accession MG913594) harbor a single nucleotide InDel in *ycf1*, which leads to a premature stop codon. A comparative sequence analysis identified eight IGRs with relatively high sequence divergence between taxa, mostly due to insertion of LSRs. In particular, *trnH*-*psbA* has been used as a plant DNA barcode [53]. The noncoding *trnH*-*psbA* spacer of *Artemisia* contains LSRs highly variable in size and sequence between and even within species, providing sufficient discriminatory power (π = 0.014) for taxon delimitation. Additionally, we identified highly diverged intergenic regions in the *Artemisia* plastome, such as *rps16-trnQ-UUG*, *trnE-UUC-rpoB*, *ndhC-trnV-UAC*, and *rbcL-accD* in LSC and *ndhF-rpl32*, *rpl32-trnL-UAG*, and *rps15-ycf1* in SSC. These regions have sufficient potential to be used as barcode markers (Additional file 2, Table S5). We anticipate that successful PCR amplification of these intergenic spacers and other polymorphic LSR loci will enable development of a single DNA marker for genotype identification below the species level. Meanwhile, consistent with a report of selected chloroplast genes in a limited number of *Artemisia* species in China [21], most protein-coding genes had low sequence divergence, with the exception of *accD*, *ycf1*, and *rps16*. These genes contained nucleotide diversity hotspots in exon (*accD* and *ycf1*) or intron (*rps16*) regions that were under weak positive selection or relaxed selective constraints. *accD* was reported to be under positive selection (ω > 1) in *A. selengensis* [38] whereas the 5′-region of the *ycf1-*coding sequence was suggested to be a plastid barcode for land plants [30]. These two genes play important roles in flowering plants. Plastid *accD* and *ycf1* are essential for plant fitness and leaf development [54] or viability [55]. Interestingly, *accD* genes in several angiosperm lineages, such as Campanulaceae and Poaceae, were lost or relocated to the nuclear genome, presumably due to endosymbiotic evolution [56]. Because the highly variable nucleotide sequences of *accD* and *ycf1* in a range of land plants are likely to be the result of environmental adaptation during evolution [57–59], they may be useful markers for plastid evolution. In this study, the conserved coding sequences flanking variable regions of *accD* and *ycf1* in *Artemisia* enabled the design of PCR primers for cross-species amplification with clear sequence polymorphisms, yielding novel *Artemisia* barcode markers with sufficient resolution to distinguish 18 (*ycf1b*), 19 (*accD*-1 k), and 21 (*accD*-1 k + *ycf1b*) plastome haplotypes from 21 *Artemisia* taxa (Table 5). This is the highest discriminatory power of chloroplast markers to identify chloroplast haplotype reported from *Artemisia* to date. Moreover, we described the utilization of *accD* and *ycf1* as molecular markers for phylogenetic analysis of the Asteraceae family. As shown in the ML tree based on 211 Asteraceae *ycf1* sequences, *ycf1* enabled tribe level resolution of the family Asteraceae (Additional file 1: Fig. S4). Phylogenetic analysis based on *accD* resulted in an ML tree showing similar topology, which enabled separation of all tribes in the family Asteraceae (Additional file 1: Fig. S5). Indeed, nucleotide sequence divergence in *accD* was concentrated in a small hotspot (Additional file 1: Fig. S2); therefore, this hotspot could serve as a universal marker for the family Asteraceae. The *ycf1b* marker, which was designed based on the consensus sequence of 144 species of 16 families [30], had a number of nucleotide mismatches in the primer binding sites for diverse Asteraceae *ycf1* genes. We designed and optimized novel primer sets for the *ycf1* and *accD* markers to ensure amplification of all Asteraceae species. Importantly, the combination of *accD* and *ycf1* has potential as a core molecular marker for Asteraceae based on its increased resolution for taxon delimitation of *Artemisia*.

Phylogenetic inference based on molecular markers that have evolved under strong pressures of natural selection would be untrustworthy [60]. According to the Ka/Ks ratio, *accD* (ω = 1.06) and *ycf1* (ω = 0.67) are assumed to have evolved under relaxation of selective constraints. Additionally, their reconstructed topologies were congruent with those inferred from different types of markers. These findings suggest that the use of *accD* and *ycf1* markers on phylogeny estimation can be potentially effective. Another strength of this study is the use of whole plastome sequences as well as 80 protein-coding genes as super-barcodes for phylogenomic inference of *Artemisia* based on maternal haplotypes. Recent progress in whole genome sequencing of *A. annua* [41] and analyses of conserved orthologs in the Asteraceae [61, 62] expanded our understanding of taxonomic relationships among *Artemisia* species based on the nuclear genome. Meanwhile, the chloroplast DNA markers developed in this study provide straightforward tools to infer evolutionary changes in the maternal lineages. The comparative analysis of complete plastome sequences and 80 protein-coding genes as super-barcodes in *Artemisia* discriminated the plastomes of most taxa and strongly supported the subgenus level classification, enabling higher-resolution analysis of relationship among taxa than prior phylogenetic studies using nuclear ITS/ETS or selected chloroplast DNA data [4, 8]. There was no difference in the tree topology between the ML and BI analyses and there was robust support for most clades, suggesting the validity of the relationships among clades and taxa (Fig. 5 and Additional file 1: Fig. S3). Interestingly, both phylogenetic trees strongly supported (100%) the monophyletic group of the subgenus *Dracunculus* consisting of *A. capillaris*, *A. dracunculus*, and *A. hallaisanensis*, whereas the subgenus *Artemisia* was paraphyletic with two divided clades. This finding was consistent with the previous molecular phylogenetic studies reporting that the subgenus *Dracunculus* was a monophyletic group and the subgnus *Artemisia* was a paraphyletic group [8, 16, 19]. In contrast, the traditional section level classification of *Artemisia* taxa [5, 6, 11, 17] had no support from clades based on the whole plastomes. A new finding is putative introgressive hybridizations between members of *A. argyi/A. argyrophylla/A. princeps* and *A. fukudo/A. nakaii.* The almost identical whole plastome sequences and sympatric distribution of these taxa suggest on-going hybridization among members of these taxa. Introgressive hybridization, combined with rapid radiation, may have caused the transfer of chloroplast haplotypes between species which might account for the inconsistency between traditional section level classification and plastome phylogeny. Molecular markers developed from nuclear genome should provide additional information to distinguish those taxa harboring the same plastome and to create reliable phylogenies of the genus *Artemisia.*

## Conclusions

The whole plastome including 80 protein-coding genes of *Artemisia* is sufficiently polymorphic to be used as super-barcodes for this genus, although they did not show 100% discriminatory power. In addition, *accD* and *ycf1* are highly polymorphic loci not only in *Artemisia* but also in the family Asteraceae; therefore, these genes have potential as universal markers for the family Asteraceae. The complete plastome investigated in this study will facilitate further research on *Artemisia* and will enhance our understanding of Asteraceae plastome evolution.

## Methods

### Plant materials and DNA extraction

A total of 19 individual samples representing 18 taxa of the genus *Artemisia* (*A. annua*, *A. apiacea*, *A. argyi*, *A. capillaris*, *A. feddei*, *A. freyniana f. discolor*, *A. fukudo*, *A. gmelinii*, *A. hallaisanensis*, *A. japonica*, *A. keiskeana*, *A. montana*, *A. nakaii*, *A. princeps*, *A. rubripes*, *A. selengensis*, *A. sieversiana*, and *A. stolonifera*) were collected in Korea. We achieved all required permits for the protected areas from the National Park Services and local governments. For *A. selengensis*, two independent samples were included. After species identification based on morphological characters, the voucher specimens were preserved in the herbarium (KB) of the National Institute of Biological Resources (Incheon, Korea). Details of the plant samples are presented in Table 1. Genomic DNAs were extracted from the dried leaves using a cetyl trimethylammonium bromide method [63] with modifications of polysaccharide precipitation by 2-propanol in the presence of 2.5 M NaCl and RNase treatment.

### Sequencing, assembly, and annotation

Illumina sequencing libraries with 500 bp inserts for 19 samples were constructed using the TruSeq DNA PCR-Free kit (Illumina, San Diego, CA, USA) and paired-end (PE) sequences (2 × 300 bp) were generated using the MiSeq platform (Illumina). The sequence data generated in this study are summarized in Additional file 2: Table S1. Additionally, Illumina reads of *A. frigida* (NCBI SRR8208356) and *A. dracunculus* (NCBI SRR8208350) were downloaded from the NCBI SRA database and were subjected to the downstream analysis. To assemble the sequence reads from each library into a circular plastome, NOVOPlasty software v2.6.6 [64] was used with *rbcL* and *rpoC2* chosen from the published plastome of *A. argyi* (GenBank accession NC030785) as seed sequences. With the draft assemblies, orientation of the SSC was determined according to its structural order in the plastome of *A. argyi* and the completeness of each assembly was verified by read mapping using Geneious Prime v2019 (Biomatters, Auckland, New Zealand) [65]. Any ambiguous regions in the draft assembles were subjected to Sanger sequencing of PCR amplicons. Genes in the plastomes were identified using Geneious Prime with a 90% similarity criterion compared to the published *Artemisia* plastomes with subsequent manual curation. tRNA genes were identified with tRNAscan-SE software [66]. All identified genes were validated by comparison with the homologs in the *Arabidopsis thaliana* plastome (GenBank accession AP000423). Circular gene maps of the *Artemisia* plastomes were generated using OGDRAW v.1.2 [67]. The plastome sequences of 11 *Artemisia* species deposited in GenBank were downloaded and re-annotated as described above. The plastomes assembled in this study have been deposited in NCBI under the accession numbers MG951482 − MG951500.

### Sequence comparison and divergence analysis

Whole plastomes of 32 *Artemisia* samples were aligned using MAFFT v7.450 [68]. Additionally, the nucleotide sequences of 80 non-redundant protein-coding genes were extracted from each plastome, concatenated into a single sequence, and aligned using the Translation Alignment tool with MAFFT in Geneious Prime. After removing gaps and poorly aligned regions using the Mask Alignment tool of Geneious Prime, the pairwise distance of 80 protein-coding genes between taxa was calculated with Geneious Prime. For each gene, multiple sequence alignment was obtained using the Translation Alignment tool with MUSCLE [69] in Geneious Prime with default options. Individual alignments were manually curated. Nucleotide diversity (average number of nucleotide differences per site between two sequences, π), number of variable sites, and PI sites were obtained using DnaSP v6 [70]. Chloroplast DNA inversions in the *Artemisia* plastomes were identified using the Mauve tool of Geneious Prime and the plastome sequence of *Nicotiana tabacum* (GenBank accession NC001879) as a reference. Ka and Ks values of the coding sequences were determined using the yn00 program in the PAML v4.9i package [71]. To identify positively selected sites in multiple sequence alignments, site models (seqtype = 1, model = 0, NSsites = 0, 1, 2, 3, 7, 8) in CodeML from PAML v4.9i or EasyCodeML v1.21 [72] were used. The unrooted ML tree of 32 *Artemisia* taxa, constructed using the MAFFT alignments of 80 protein-coding genes and RAxML v8.2.9 [73], was used as an input tree. The amino acid sites under positive selection were detected based on the results of a posterior BEB analysis with a probability threshold of 0.95. SSRs with a unit length of 2–6 bp repeated at least three times were surveyed across the 32 *Artemisia* whole plastomes using Phobos v3.3.12 in Geneious Prime. LSRs were identified using Repeat Finder v1.0.1 in Geneious Prime and their structures and inter-taxon polymorphisms were manually curated. LSR was defined as 11–100 bp with spacers no longer than 50 bp and 100% identity between sequences. Both direct tandem and hairpin repeats were considered, and palindromic structures were analyzed. LSRs located in SSR regions were excluded to reduce redundancy.

### Phylogenetic analysis

Phylogenetic trees based on 80 protein-coding genes of 32 *Artemisia* plastomes were generated by the ML and BI methods using RAxML v8.2.9 [73] and MrBayes v3.2.7 [74], respectively, with *Aster spathulifolius* (GenBank accession NC027434) as an outgroup. For ML analysis, partitioning of protein-coding genes was carried out using PartitionFinder2 with a greedy algorithm [75] and unlinked branchlengths parameter. The GTR + G model for protein-coding genes and the GRT + G + I model for whole plastomes was chosen as the best-fit DNA substitution model according to the Akaike Information Criterion correction in ModelTest-NG v0.1.6 [76]. ML trees were constructed using rapid bootstrapping and search for the best-scoring ML tree option of RAxML v8.2.9 [73] with 1000 bootstrap replicates. To choose appropriate outgroup taxa, the species from several tribes of the Asteraceae, including *Cynara humilis* in Cardueae, *Helianthus annuus* in Helentheae, *Aster spathulifolius* in Astereae, and *Chrysanthemun boreale* and *C. indicum* in Anthemideae, were analyzed together with *Artemisia* taxa. Among them, *A. spathulifolius* (GenBank accession NC027434) was chosen as an outgroup species due to its distinct separation from the ingroup taxa. For Bayesian analysis, the Markov chain Monte Carlo (MCMC) algorithm was applied for 11 million generations with four heated chains and sampling of trees every 1000 generations. The first 25% of trees were discarded as burn-in. The average standard deviation of split frequencies was below 0.01 after 800,000 generations and 0.0028 at the final generation. The Potential Scale Reduction Factor, a convergence diagnostic, was 1.001 on average. The posterior distribution was evaluated using Tracer v1.7 [77]. On the trace plot of log-likelihood, a good sign of MCMC convergence was shown before 10% proportion of burn-in. The consensus trees were finally edited using the MEGA7 software [78].

### Development of Asteraceae markers

Complete plastomes of Asteraceae species were downloaded from NCBI non-redundant DNA database. In the case of redundant sequences available for the same accession, further analysis collapsed them into one representative plastome. The coding sequences of *accD* and *ycf1* from 219 plastomes, identified using BLASTN (BLAST+ v2.10.0) search, were extracted and aligned using MUSCLE [69]. Sequences shorter than 1 kb for *accD* and 1.5 kb for *ycf1* were excluded. The sequence alignments of 212 *accD* and 211 *ycf1* genes were further refined by translation align in Geneious Prime. For the combinations of these two genes, aligned sequences of *accD* and *ycf1* were concatenated. A phylogenetic tree of Asteraceae *accD* and *ycf1* was constructed by the ML method using RAxML software with the GTRGAMMA model and 1000 bootstrap replications. The barcode PCR primers were manually designed based on the consensus sequences generated from the multiple sequence alignments at a threshold level of 95%. In silico PCR analysis was conducted using FastPCR software v6.0 [79].

## Supplementary information

**Additional file 1 : Figure S1 to S7. Fig. S1.** Comparison of the IR border regions of the Asteraceae plastomes. **Fig. S2.** Multiple alignments of *accD* coding sequences in the 32 *Artemisia* plastomes showing hotspots of nucleotide sequence diversity. Eight positively selected amino acid substitutions are indicated by red triangles. The core hotspot of 276 bp in length (616–963 bp over the gapped alignment) is indicated by arrows. **Fig. S3.** A ML tree based on the whole plastomes of 32 *Artemisia* taxa. Bootstrap values are indicated on the nodes. Colored lines and braces at the right of the tree indicate section and subgenus names of *Artemisia*, respectively, that include taxa. **Fig. S4.** A ML tree of *ycf1* in the Asteraceae family. Taxa belonging to the same supertribe or subfamily are grouped. **Fig. S5.** A ML tree of *accD* in the Asteraceae family. Taxa belonging to the same supertribe or subfamily are grouped. **Fig. S6**. Performances of *accD* and *ycf1* in identifying the Asteraceae taxa using BLAST search. Hits with 100% identity were counted into two categories, unique hit and cross-hit to other species or tribe(s). **Fig. S7.** A ML tree based on the *accD*-1 k + *ycf1b* marker sequences of 32 *Artemisia* taxa. Bootstrap values are indicated on the nodes.

**Additional file 2 : Tables S1 to S8. Table S1.** Sample information and statistics of the Illumina PE sequence data of *Artemisia* taxa. **Table S2.** Gene contents of the *Artemisia* plastomes. **Table S3.** Pairwise nucleotide similarity matrix of the 32 *Artemisia* plastomes. **Table S4.** Highly variable regions among the 32 *Artemisia* plastomes. **Table S5**. Nucleotide diversity and polymorphism of 11 highly diverged regions in the *Artemisia* plastome. **Table S6.** Likelihood ratio tests to identify positively selected sites within the *accD* and *ycf1* coding sequences across the 32 *Artemisia* plastomes. **Table S7.** Polymorphic LSRs identified in the 32 *Artemisia* plastomes. **Table S8.** In silico PCR analysis of the accD-Asteraceae and ycf1b-Asteraceae markers.

## Data Availability

The assembled sequences described in this study have been deposited in the National Center for Biotechnology and Information (NCBI) under the accessions as summarized in Table 1.

## Abbreviations

BEB: Bayes empirical Bayes
BI: Bayesian Inference
bp: base pairs
ETS: External transcribed spacer
Gb: Gigabases
IGR: Intergenic region
InDel: Insertion or deletion
IR: Inverted repeat
ITS: Internal transcribed spacer
Ka: Non-synonymous substitution rate
kb: kilobases
Ks: Synonymous substitution rate
LSC: Long single copy
LSR: Long sequence repeat
MCMC: Markov chain Monte Carlo
ML: Maximum likelihood
NCBI: National Center for Biotechnology Information
NGS: Next generation sequencing
ω: Ka/Ks
π: nucleotide diversity
PCR: Polymerase chain reaction
PI: Parsimony informative
rRNA: ribosomal RNA
SRA: Sequence Read Archive
SSC: Short single copy
SSR: Simple sequence repeat
tRNA: transfer RNA
