# Supplementary material for: Comparative chloroplast genome analysis of Artemisia (Asteraceae) in East Asia: insights into evolutionary divergence and phylogenomic implications
Source: BMC Genomics. 2020 Jun 22;21:415. doi: 10.1186/s12864-020-06812-7 (PMC7310033; doi:10.1186/s12864-020-06812-7)
Supplement: Supplementary file 1 — Additional file 1 : Figure S1 to S7. Fig. S1. Comparison of the IR border regions of the Asteraceae plastomes. Fig. S2. Multiple alignments of accD coding sequences in the 32 Artemisia plastomes showing hotspots of nucleotide sequence diversity. Eight positively selected amino acid substitutions are indicated by red triangles. The core hotspot of 276 bp in length (616–963 bp over the gapped alignment) is indicated by arrows. Fig. S3. A ML tree based on the whole plastomes of 32 Artemisia taxa. Bootstrap values are indicated on the nodes. Colored lines and braces at the right of the tree indicate section and subgenus names of Artemisia, respectively, that include taxa. Fig. S4. A ML tree of ycf1 in the Asteraceae family. Taxa belonging to the same supertribe or subfamily are grouped. Fig. S5. A ML tree of accD in the Asteraceae family. Taxa belonging to the same supertribe or subfamily are grouped. Fig. S6. Performances of accD and ycf1 in identifying the Asteraceae taxa using BLAST search. Hits with 100% identity were counted into two categories, unique hit and cross-hit to other species or tribe(s). Fig. S7. A ML tree based on the accD-1 k + ycf1b marker sequences of 32 Artemisia taxa. Bootstrap values are indicated on the nodes. [file 12864_2020_6812_MOESM1_ESM.pptx]

## Slide 1
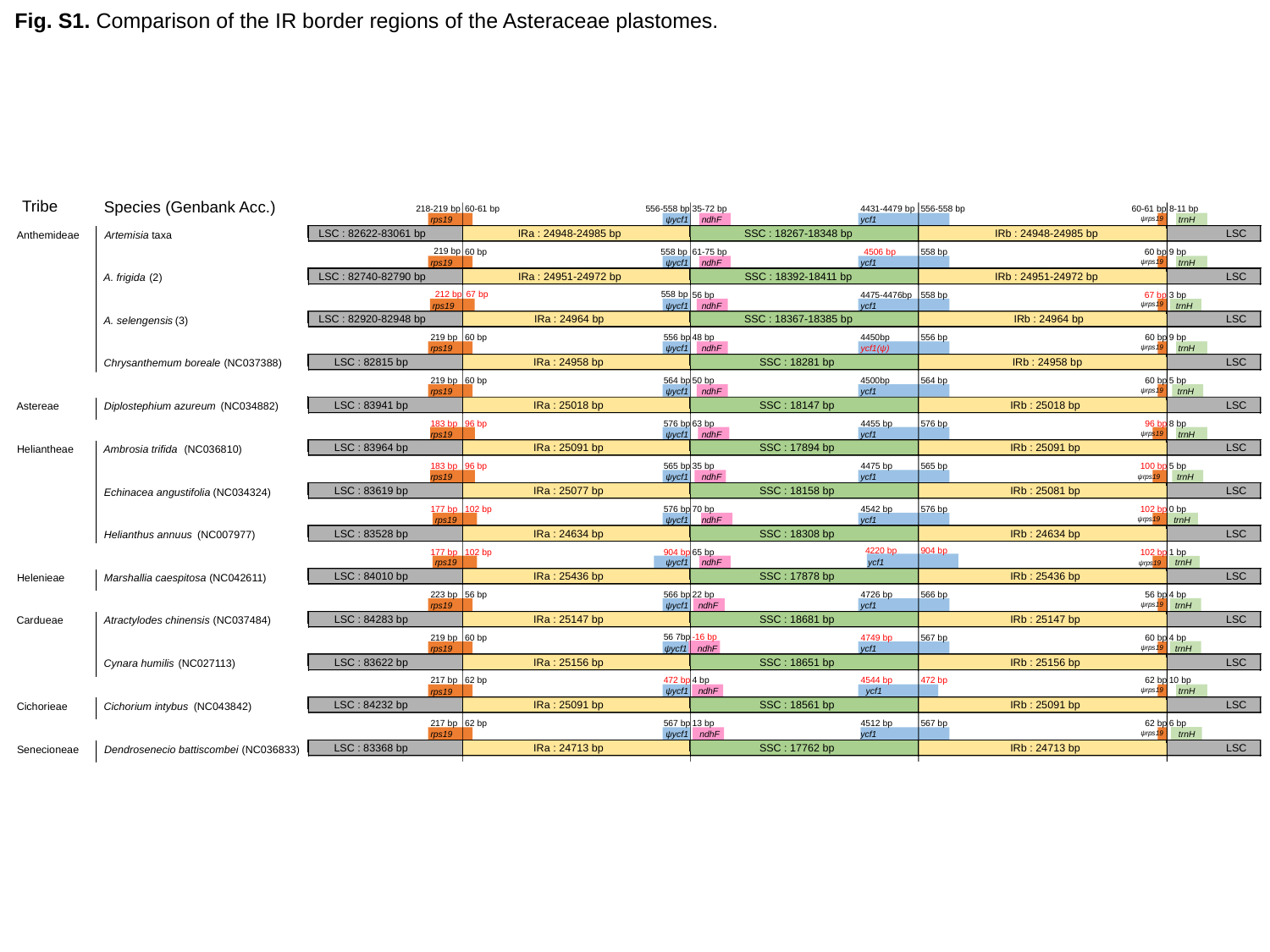

Fig. S1. Comparison of the IR border regions of the Asteraceae plastomes.
Tribe
Species (Genbank Acc.)
218-219 bp
60-61 bp
556-558 bp
35-72 bp
4431-4479 bp
556-558 bp
60-61 bp
8-11 bp
rps19
ψycf1
ndhF
ycf1
ψrps19
trnH
LSC : 82622-83061 bp
IRa : 24948-24985 bp
SSC : 18267-18348 bp
IRb : 24948-24985 bp
LSC
Anthemideae
Artemisia taxa
219 bp
558 bp
60 bp
61-75 bp
4506 bp
558 bp
60 bp
9 bp
rps19
ψycf1
ndhF
ycf1
ψrps19
trnH
LSC : 82740-82790 bp
IRa : 24951-24972 bp
SSC : 18392-18411 bp
IRb : 24951-24972 bp
LSC
A. frigida
 (2)
212 bp
67 bp
558 bp
56 bp
4475-4476bp
558 bp
67 bp
3 bp
rps19
ψycf1
ndhF
ycf1
ψrps19
trnH
LSC : 82920-82948 bp
IRa : 24964 bp
SSC : 18367-18385 bp
IRb : 24964 bp
LSC
A. selengensis
 (3)
219 bp
60 bp
556 bp
48 bp
4450bp
556 bp
60 bp
9 bp
rps19
ψycf1
ndhF
ycf1(ψ)
ψrps19
trnH
LSC : 82815 bp
IRa : 24958 bp
SSC : 18281 bp
IRb : 24958 bp
LSC
Chrysanthemum boreale
(NC037388)
219 bp
60 bp
564 bp
50 bp
4500bp
564 bp
60 bp
5 bp
rps19
ψycf1
ndhF
ycf1
ψrps19
trnH
LSC : 83941 bp
IRa : 25018 bp
SSC : 18147 bp
IRb : 25018 bp
LSC
Astereae
Diplostephium azureum
(NC034882)
183 bp
96 bp
576 bp
63 bp
4455 bp
576 bp
96 bp
8 bp
rps19
ψycf1
ndhF
ycf1
ψrps19
trnH
LSC : 83964 bp
IRa : 25091 bp
SSC : 17894 bp
IRb : 25091 bp
LSC
Heliantheae
Ambrosia trifida
(NC036810)
183 bp
96 bp
565 bp
35 bp
4475 bp
565 bp
100 bp
5 bp
ψrps19
rps19
ψycf1
ndhF
ycf1
trnH
LSC : 83619 bp
IRa : 25077 bp
SSC : 18158 bp
IRb : 25081 bp
LSC
Echinacea angustifolia
(NC034324)
177 bp
102 bp
576 bp
70 bp
4542 bp
576 bp
102 bp
0 bp
ψrps19
rps19
ψycf1
ndhF
ycf1
trnH
LSC : 83528 bp
IRa : 24634 bp
SSC : 18308 bp
IRb : 24634 bp
LSC
Helianthus annuus
(NC007977)
4220 bp
904 bp
177 bp
102 bp
904 bp
65 bp
102 bp
1 bp
ψrps19
rps19
ψycf1
ndhF
ycf1
trnH
LSC : 84010 bp
IRa : 25436 bp
SSC : 17878 bp
IRb : 25436 bp
LSC
Helenieae
Marshallia caespitosa
(NC042611)
223 bp
56 bp
566 bp
22 bp
4726 bp
566 bp
56 bp
4 bp
rps19
ψycf1
ndhF
ycf1
ψrps19
trnH
LSC : 84283 bp
IRa : 25147 bp
SSC : 18681 bp
IRb : 25147 bp
LSC
Cardueae
Atractylodes chinensis
(NC037484)
56 7bp
-16 bp
219 bp
60 bp
4749 bp
567 bp
60 bp
4 bp
rps19
ψycf1
ndhF
ycf1
ψrps19
trnH
LSC : 83622 bp
IRa : 25156 bp
SSC : 18651 bp
IRb : 25156 bp
LSC
Cynara humilis
(NC027113)
217 bp
62 bp
472 bp
4 bp
4544 bp
472 bp
62 bp
10 bp
rps19
ψycf1
ndhF
ycf1
ψrps19
trnH
LSC : 84232 bp
IRa : 25091 bp
SSC : 18561 bp
IRb : 25091 bp
LSC
Cichorieae
Cichorium intybus
(NC043842)
217 bp
62 bp
567 bp
13 bp
4512 bp
567 bp
62 bp
6 bp
rps19
ψycf1
ndhF
ycf1
ψrps19
trnH
LSC : 83368 bp
IRa : 24713 bp
SSC : 17762 bp
IRb : 24713 bp
LSC
Senecioneae
Dendrosenecio battiscombei
(NC036833)

## Slide 2
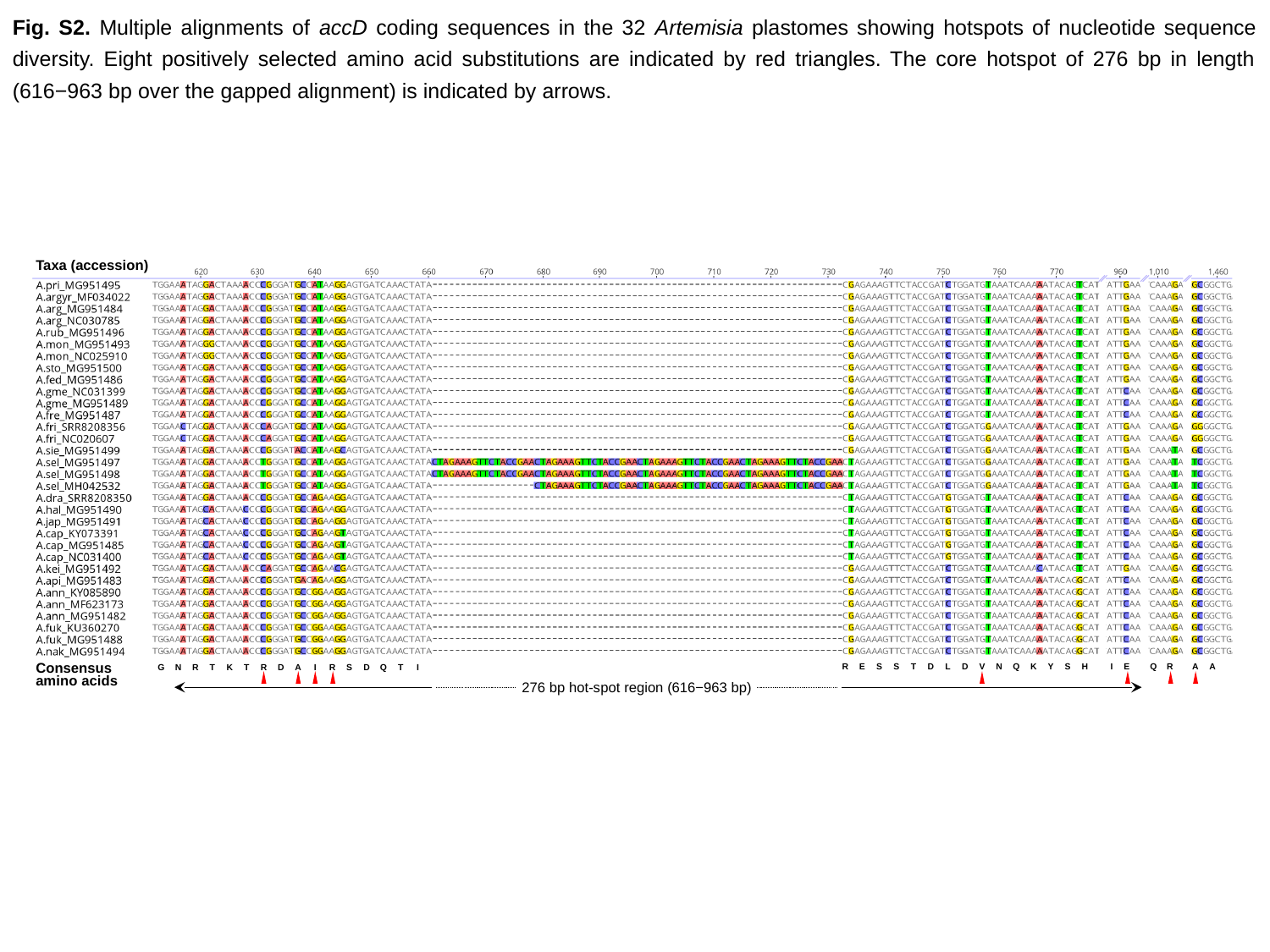

Fig. S2. Multiple alignments of accD coding sequences in the 32 Artemisia plastomes showing hotspots of nucleotide sequence diversity. Eight positively selected amino acid substitutions are indicated by red triangles. The core hotspot of 276 bp in length (616−963 bp over the gapped alignment) is indicated by arrows.
Taxa (accession)
I
E
Q
R
A
A
R
E
S
S
T
D
L
D
V
N
Q
K
Y
S
H
G
N
R
T
K
T
R
D
A
I
R
S
D
Q
T
I
Consensus
amino acids
276 bp hot-spot region (616−963 bp)

## Slide 3
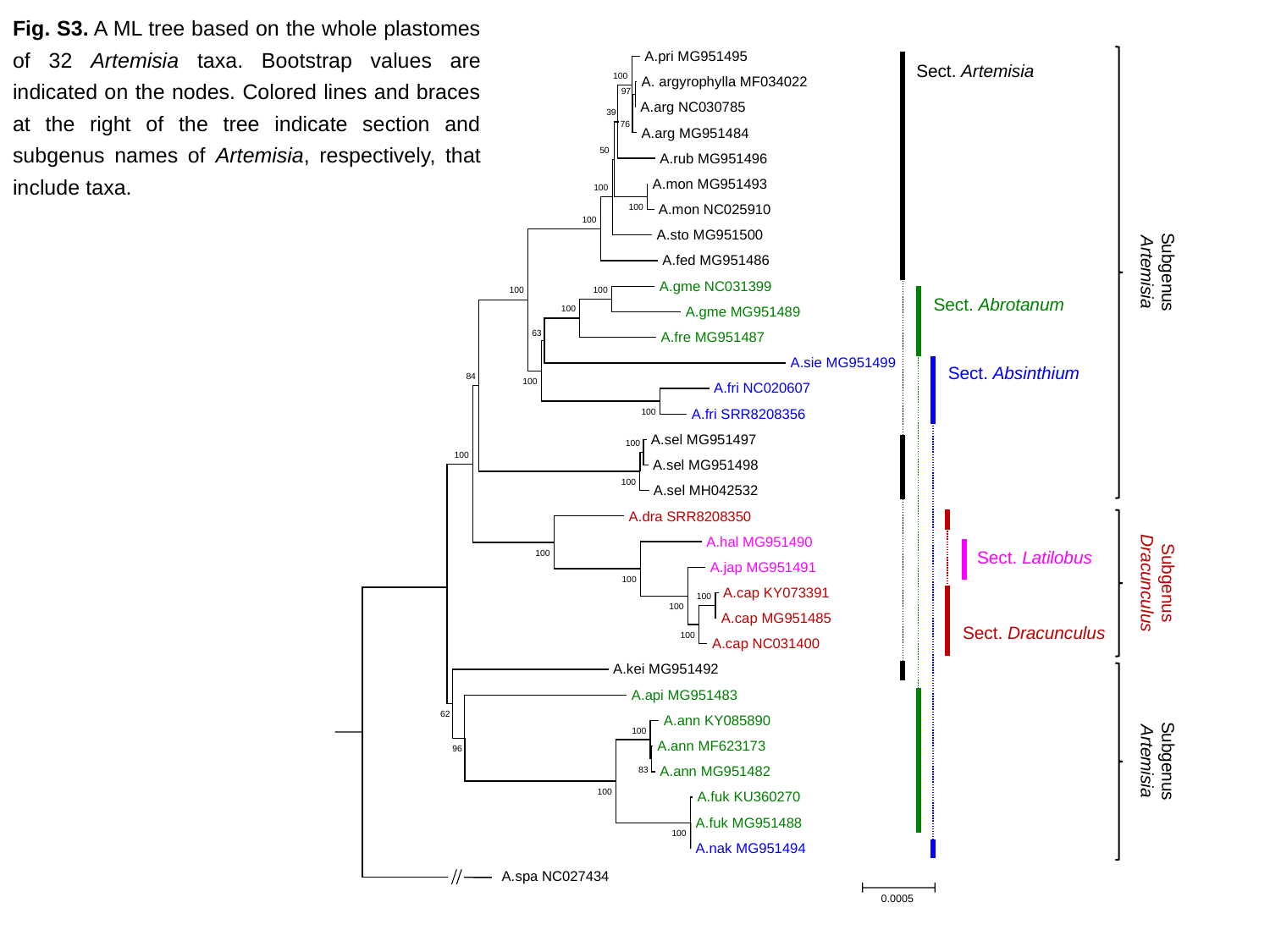

Fig. S3. A ML tree based on the whole plastomes of 32 Artemisia taxa. Bootstrap values are indicated on the nodes. Colored lines and braces at the right of the tree indicate section and subgenus names of Artemisia, respectively, that include taxa.
 A.pri MG951495
100
 A. argyrophylla MF034022
97
 A.arg NC030785
39
76
 A.arg MG951484
50
 A.rub MG951496
 A.mon MG951493
100
 A.mon NC025910
100
100
 A.sto MG951500
 A.fed MG951486
 A.gme NC031399
100
100
 A.gme MG951489
100
63
 A.fre MG951487
 A.sie MG951499
84
100
 A.fri NC020607
 A.fri SRR8208356
100
 A.sel MG951497
100
100
 A.sel MG951498
100
 A.sel MH042532
 A.dra SRR8208350
 A.hal MG951490
100
 A.jap MG951491
100
 A.cap KY073391
100
100
 A.cap MG951485
100
 A.cap NC031400
 A.kei MG951492
 A.api MG951483
62
 A.ann KY085890
100
 A.ann MF623173
96
 A.ann MG951482
83
100
 A.fuk KU360270
 A.fuk MG951488
100
 A.nak MG951494
 A.spa NC027434
Sect. Artemisia
Sect. Abrotanum
Sect. Absinthium
Sect. Latilobus
Sect. Dracunculus
0.0005
Subgenus
Artemisia
Subgenus
Dracunculus
Subgenus
Artemisia

## Slide 4
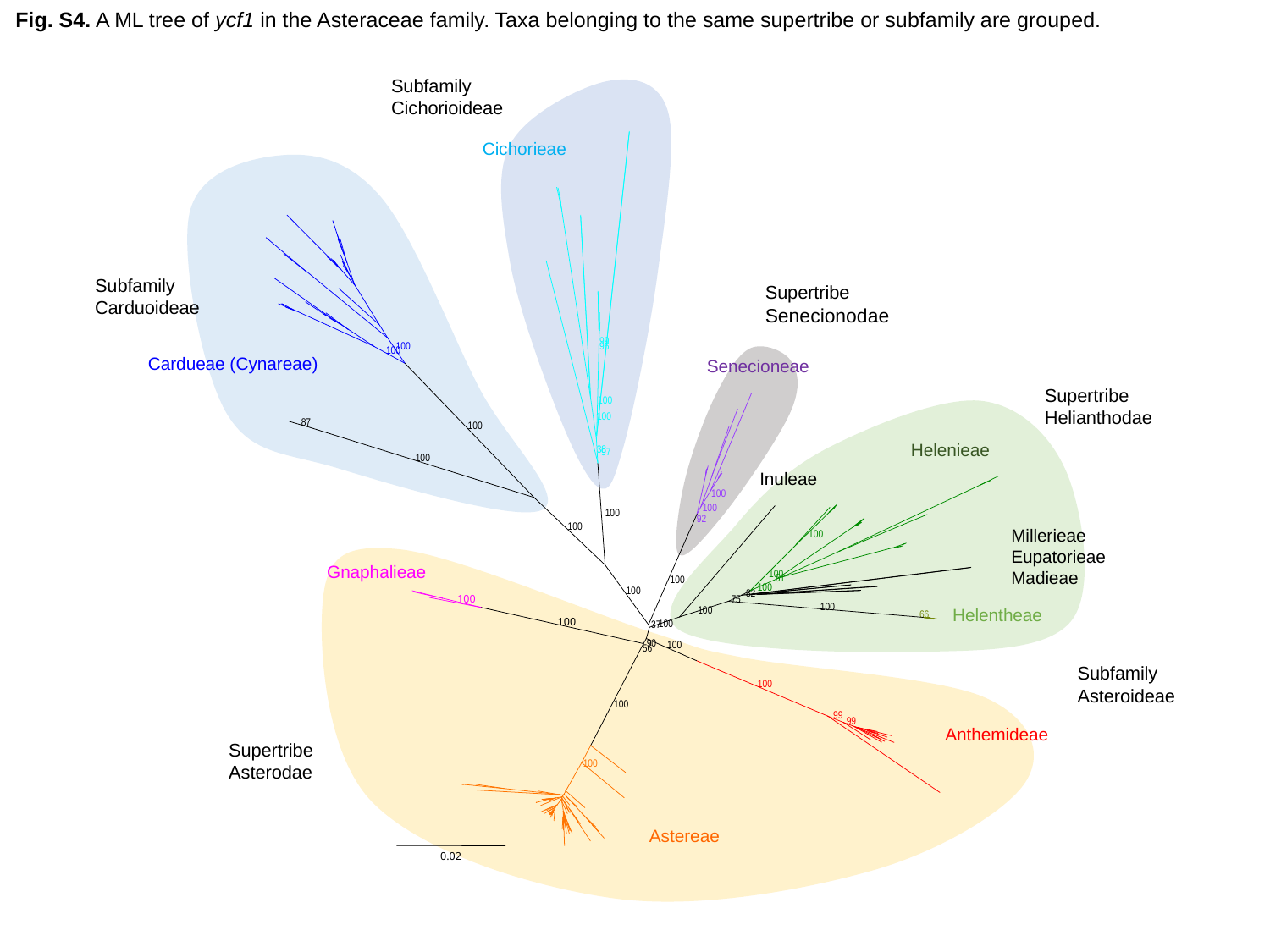

Fig. S4. A ML tree of ycf1 in the Asteraceae family. Taxa belonging to the same supertribe or subfamily are grouped.
Subfamily
Cichorioideae
Cichorieae
 99
 96
 100
 100
 100
 100
 87
 100
 38
 97
 100
 100
 100
 100
 92
 100
 100
 100
 51
 100
 100
 100
 82
 100
 75
 100
 100
 66
 100
 100
 37
 90
 100
 56
 100
 100
 99
 99
 100
0.02
Subfamily
Carduoideae
Supertribe
Senecionodae
Cardueae (Cynareae)
Senecioneae
Supertribe
Helianthodae
Helenieae
Inuleae
Millerieae
Eupatorieae
Madieae
Gnaphalieae
Helentheae
Subfamily
Asteroideae
Anthemideae
Supertribe
Asterodae
Astereae

## Slide 5
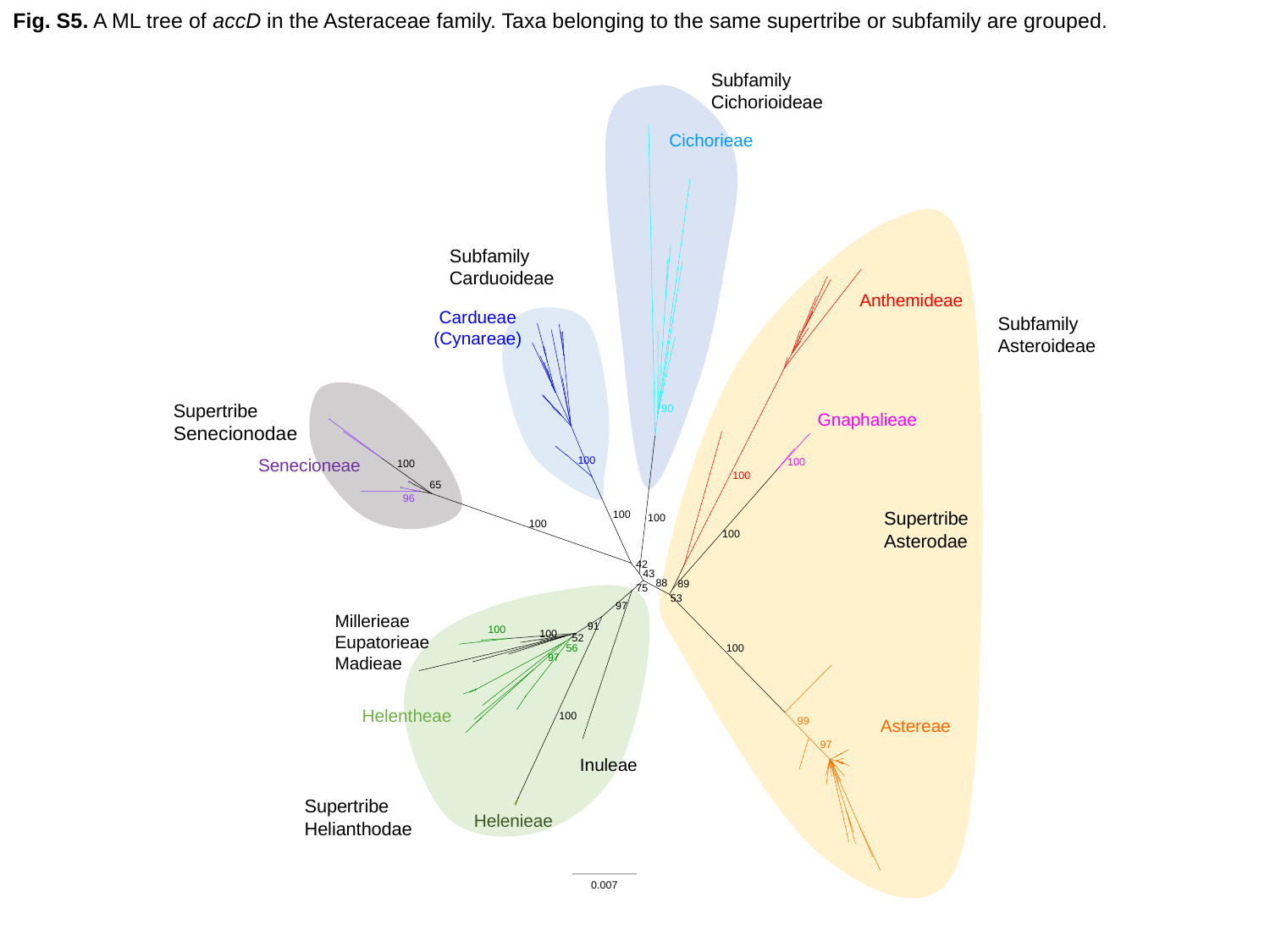

Fig. S5. A ML tree of accD in the Asteraceae family. Taxa belonging to the same supertribe or subfamily are grouped.
Subfamily
Cichorioideae
 90
 100
 100
 100
 100
 100
 100
 100
 100
 42
 43
 88
 89
 75
 53
 97
 91
 52
 100
 100
 99
 97
 65
 96
 100
 100
 56
 97
0.007
Cichorieae
Subfamily
Carduoideae
Anthemideae
Cardueae
(Cynareae)
Subfamily
Asteroideae
Supertribe
Senecionodae
Gnaphalieae
Senecioneae
Supertribe
Asterodae
Millerieae
Eupatorieae
Madieae
Helentheae
Astereae
Inuleae
Supertribe
Helianthodae
Helenieae

## Slide 6
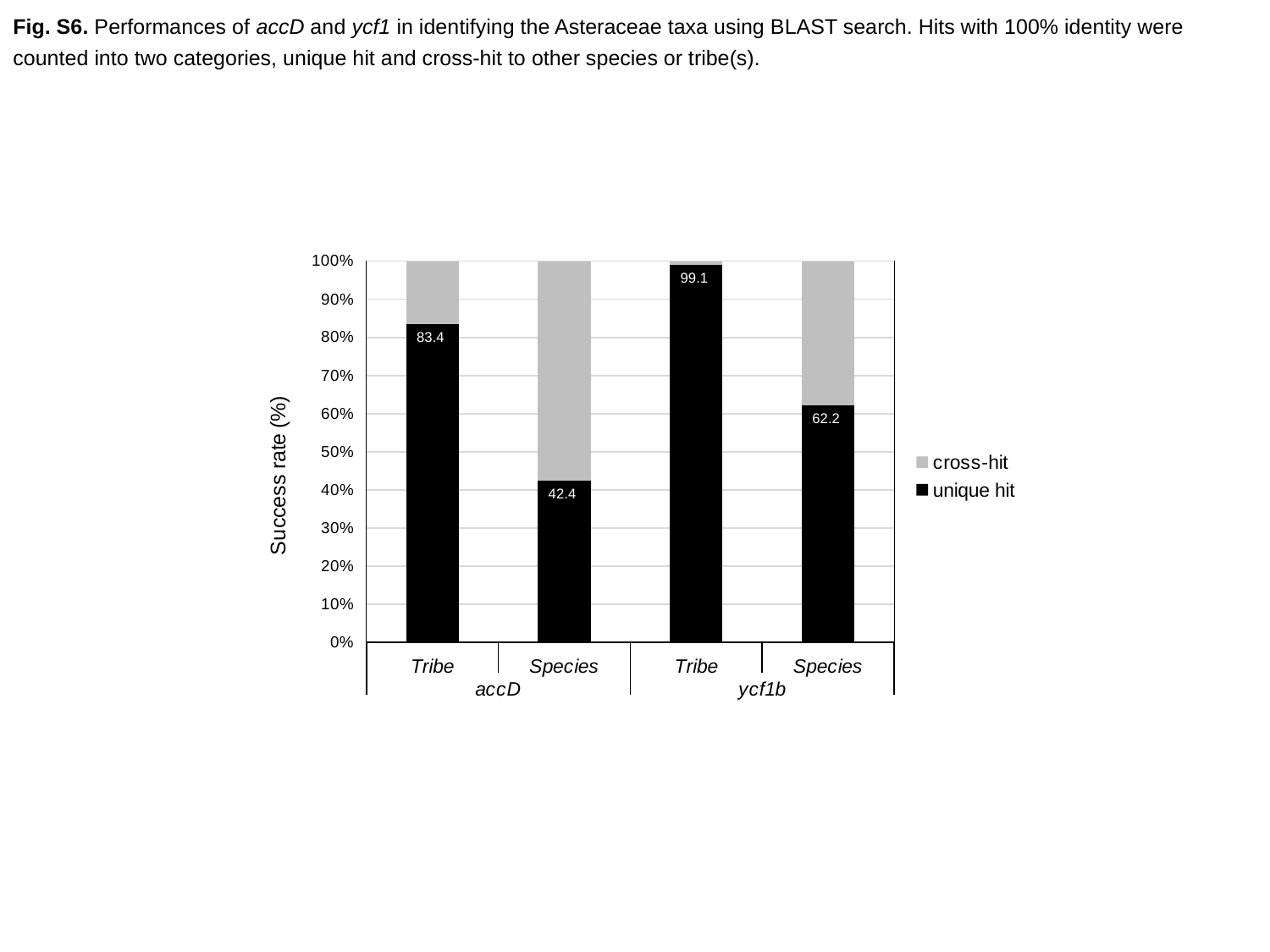

Fig. S6. Performances of accD and ycf1 in identifying the Asteraceae taxa using BLAST search. Hits with 100% identity were counted into two categories, unique hit and cross-hit to other species or tribe(s).
### Chart
| Category | unique hit | cross-hit |
|---|---|---|
| Tribe | 83.4061135371179 | 16.593886462882097 |
| Species | 42.35807860262008 | 57.64192139737992 |
| Tribe | 99.11111111111111 | 0.8888888888888888 |
| Species | 62.22222222222222 | 37.77777777777778 |

## Slide 7
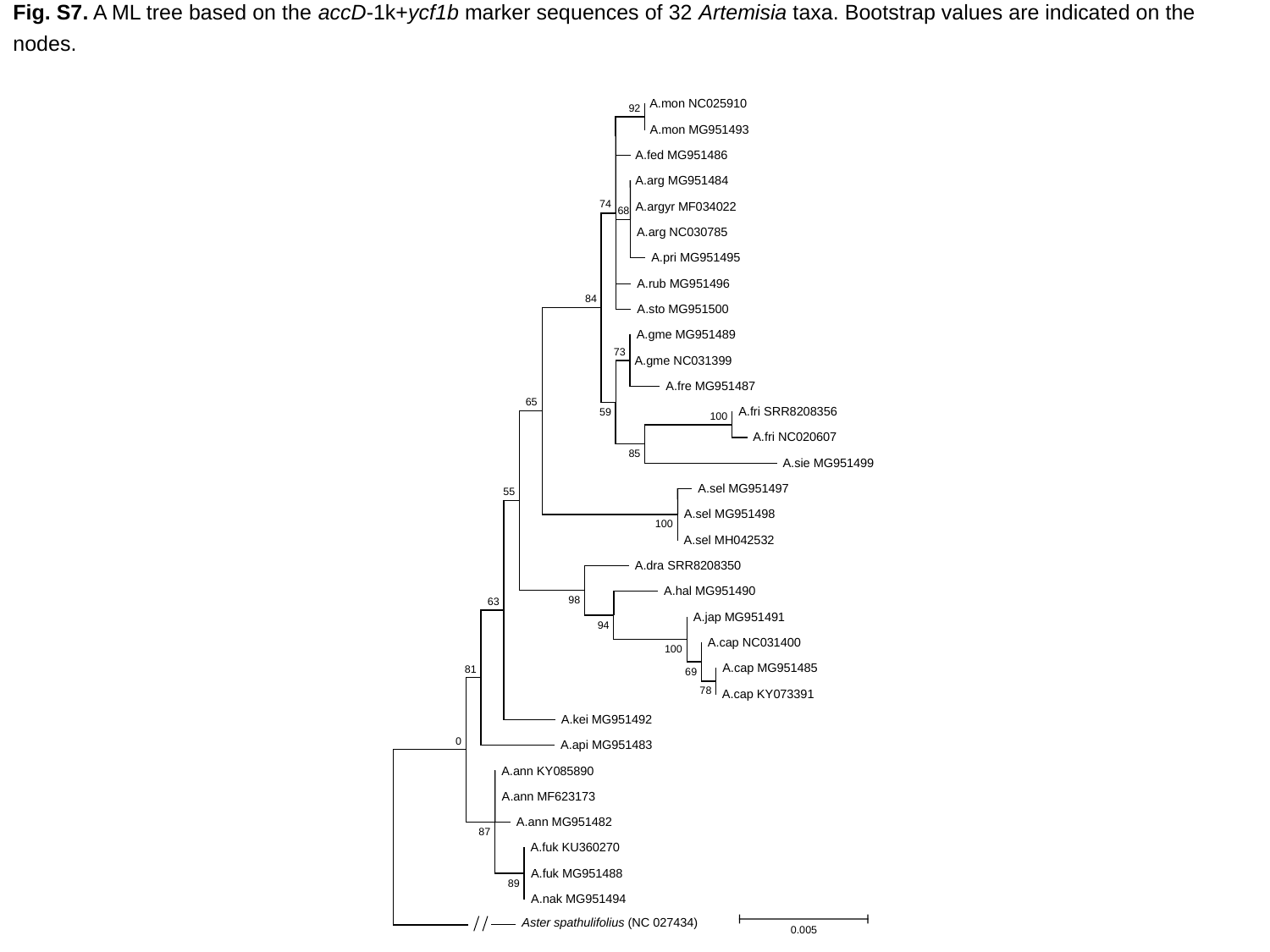

Fig. S7. A ML tree based on the accD-1k+ycf1b marker sequences of 32 Artemisia taxa. Bootstrap values are indicated on the nodes.
 A.mon NC025910
92
 A.mon MG951493
 A.fed MG951486
 A.arg MG951484
74
 A.argyr MF034022
68
 A.arg NC030785
 A.pri MG951495
 A.rub MG951496
84
 A.sto MG951500
 A.gme MG951489
73
 A.gme NC031399
 A.fre MG951487
65
 A.fri SRR8208356
59
100
 A.fri NC020607
85
 A.sie MG951499
 A.sel MG951497
55
 A.sel MG951498
100
 A.sel MH042532
 A.dra SRR8208350
 A.hal MG951490
98
63
 A.jap MG951491
94
 A.cap NC031400
100
 A.cap MG951485
81
69
78
 A.cap KY073391
 A.kei MG951492
0
 A.api MG951483
 A.ann KY085890
 A.ann MF623173
 A.ann MG951482
87
 A.fuk KU360270
 A.fuk MG951488
89
 A.nak MG951494
0.005
 Aster spathulifolius (NC 027434)
