# Supplementary material for: Comparative chloroplast genome analysis of Artemisia (Asteraceae) in East Asia: insights into evolutionary divergence and phylogenomic implications
Source: BMC Genomics. 2020 Jun 22;21:415. doi: 10.1186/s12864-020-06812-7 (PMC7310033; doi:10.1186/s12864-020-06812-7)
Supplement: Supplementary file 2 — Additional file 2 : Tables S1 to S8. Table S1. Sample information and statistics of the Illumina PE sequence data of Artemisia taxa. Table S2. Gene contents of the Artemisia plastomes. Table S3. Pairwise nucleotide similarity matrix of the 32 Artemisia plastomes. Table S4. Highly variable regions among the 32 Artemisia plastomes. Table S5. Nucleotide diversity and polymorphism of 11 highly diverged regions in the Artemisia plastome. Table S6. Likelihood ratio tests to identify positively selected sites within the accD and ycf1 coding sequences across the 32 Artemisia plastomes. Table S7. Polymorphic LSRs identified in the 32 Artemisia plastomes. Table S8. In silico PCR analysis of the accD-Asteraceae and ycf1b-Asteraceae markers. [file 12864_2020_6812_MOESM2_ESM.docx]

**Table S1.** Sample information and statistics of the Illumina PE sequence data of *Artemisia* taxa

| Section | Scientific name | Voucher^1^ | Illumina read statistics^2^ | | |
| --- | --- | --- | --- | --- | --- |
|  |  |  | Total base (Mb) | Q20  (%) | Q30  (%) |
| *Abrotanum* | *A. annua* | NIBRVP0000595661 | 12,305 | 83.4 | 75.3 |
|  | *A. apiacea* | NIBRVP0000538751 | 10,251 | 86.1 | 78.6 |
|  | *A. freyniana* f. *discolor* | NIBRVP0000538858 | 10,597 | 75.9 | 67.2 |
|  | *A. fukudo* | NIBRVP0000597993 | 10,768 | 91.1 | 83.5 |
|  | *A. gmelinii* | NIBRVP0000592776 | 12,993 | 89.1 | 81.4 |
| *Absinthium* | *A. nakaii* | NIBRVP0000598807 | 10,955 | 88.1 | 80.1 |
|  | *A. sieversiana* | NIBRVP0000592824 | 10,805 | 82.3 | 74.2 |
| *Artemisia* | *A. argyi* | NIBRVP0000592833 | 9,377 | 86.1 | 79.3 |
|  | *A. feddei* | NIBRVP0000592740 | 8,254 | 87.1 | 78.8 |
|  | *A. keiskeana* | NIBRVP0000592791 | 9,829 | 89.4 | 82.4 |
|  | *A. montana* | NIBRVP0000627850 | 11,035 | 88.1 | 79.8 |
|  | *A. princeps* | NIBRVP0000592810 | 10,139 | 91.7 | 86.0 |
|  | *A. rubripes* | NIBRVP0000592774 | 10,783 | 88.9 | 82.2 |
|  | *A. selengensis* | NIBRVP0000538775 | 12,796 | 88.9 | 80.7 |
|  | *A. selengensis* | NIBRVP0000595650 | 10,317 | 86.6 | 78.8 |
|  | *A. stolonifera* | NIBRVP0000592785 | 9,499 | 88.3 | 81.0 |
| *Dracunculus* | *A. capillaris* | NIBRVP0000592735 | 12,747 | 83.8 | 75.1 |
| *Latilobus* | *A. hallaisanensis* | NIBRVP0000538771 | 7,885 | 77.7 | 68.1 |
|  | *A. japonica* | NIBRVP0000592828 | 10,087 | 82.4 | 74.5 |

^1^Deposited in the herbarium (KB) of the National Institute of Biological Resources (Incheon, Korea).

^2^Paired-end reads (2×300 bp) were generated using Illumina MiSeq platform.

**Table S2.** Gene contents of the *Artemisia* plastomes

| Group | | Name of genes* | Numbers |
| --- | --- | --- | --- |
| RNA genes | rRNAs | rrn4.5(×2), rrn5(×2), rrn16(×2), rrn23(×2) | 8 |
|  | tRNAs | trnA-UGC^a^(×2), trnC-GCA, trnD-GUC, trnE-UUC, trnF-GAA, trnfM-CAU, trnG-GCC, trnG-UCC^a^, trnH-GUG, trnI-CAU(×2), trnI-GAU^a^(×2), trnK-UUU^a^, trnL-CAA(×2), trnL-UAA^a^, trnL-UAG, trnM-CAU, trnN-GUU(×2), trnP-UGG, trnQ-UUG, trnR-ACG(×2), trnR-UCU, trnS-GCU, trnS-GGA, trnS-UGA, trnT-GGU, trnT-UGU, trnV-GAC(×2), trnV-UAC^a^, trnW-CCA, trnY-GUA | 37 |
| Protein genes | Photosystem I | psaA, psaB, psaC, psaI, psaJ | 5 |
|  | Photosystem II | psbA, psbB, psbC, psbD, psbE, psbF, psbH, psbI, psbJ, psbK, psbL, psbM, psbN, psbT, psbZ | 15 |
|  | Cytochrome | petA, petB^a^,petD^a^,petG,petL,petN | 6 |
|  | ATP synthase | atpA, atpB, atpE, atpF^a^,atpH,atpI | 6 |
|  | Rubisco | rbcL | 1 |
|  | NADH-dehydrogenease | ndhA^a^,ndhB^a^(×2), ndhC, ndhD, ndhE, ndhF,  ndhG, ndhH, ndhI, ndhJ, ndhK | 12 |
|  | ATP-dependent protease subunit P | clpP^a^ | 1 |
|  | Chloroplast envelope membrane protein | cemA | 1 |
| Ribosomal  proteins | large units | rpl2^a^(×2), rpl14, rpl16^a^, rpl20, rpl22, rpl23(×2), rpl32, rpl33, rpl36 | 10 |
|  | small units | rps2, rps3, rps4, rps7(×2), rps8, rps11, rps12^a^(×2), rps14, rps15, rps16^a^, rps18, rps19 | 14 |
| Transcription | RNA polymerase | rpoA, rpoB, rpoC1^a^, rpoC2 | 4 |
| /translation | Initiation factor | infA | 1 |
|  | Miscellaneous proteins | accD, ccsA, matK | 3 |
|  | Hypothetical proteins &  Conserved reading frame | ycf1, ycf2(×2), ycf3^a^, ycf4, ycf15(×2) | 7 |
| Total |  |  | 132 |
| ^*^(×2), duplicated genes; ^a^genes containing intron(s). | | | |

**Table S3.** Pairwise nucleotide similarity matrix of the 32 *Artemisia* plastomes

^*^Upper half of the matrix is pairwise similarities between the whole plastomes (red/blue heat map) and the lower half (red/green heat map) is those of the coding sequences.

**Table S4.** Highly variable regions among the 32 *Artemisia* plastomes

| Locus | Midpoint of the most variable window (bp) | Variable region | Peak π | Structural region |
| --- | --- | --- | --- | --- |
| *trnH-psbA* | 742 | 110−1242 | 0.00620 | LSC |
| *rps16* | 6349 | 5624−7028 | 0.00738 | LSC |
| *rps16-trnQ-UUG* | 6881 | 6085−7677 | 0.00755 | LSC |
| *trnE-UUC-rpoB* | 12641 | 12130−13191 | 0.00609 | LSC |
| *ndhC-trnV-UAC* | 51411 | 50752−52082 | 0.00706 | LSC |
| *rbcL-accD* | 58110 | 57472−58782 | 0.00831 | LSC |
| *accD* | 58651 | 57811−59482 | 0.00804 | LSC |
| *ndhF-rpl32* | 112530 | 111436−113761 | 0.00972 | SSC |
| *rpl32-trnL-UAG* | 113642 | 112634−114492 | 0.00784 | SSC |
| *rps15-ycf1* | 124028 | 123457−124582 | 0.00731 | SSC |
| *ycf1* | 126107 | 123674−128494 | 0.00847 | SSC |

**Table S5.** Nucleotide diversity and polymorphism of 11 highly diverged regions in the *Artemisia* plastome

|  | Intergenic spacer region^1^ | | | | | | | |  | Genic region (exon and intron) | | | |
| --- | --- | --- | --- | --- | --- | --- | --- | --- | --- | --- | --- | --- | --- |
|  | *trnH- psbA* | *ndhC-*  *trnV-UAC* | *rbcL-accD* | *rps16-*  *trnQ-UUG* | *trnE-UUC-*  *rpoB* | *rps15-ycf1* | *ndhF-rpl32* | *rpl32-*  *trnL-UAG* |  | *rps16*^2^ | *accD*^3^ | *ycf1b*^3^ |  |
| Genome location | LSC | LSC | LSC | LSC | LSC | SSC | SSC | SSC |  | LSC | LSC | SSC |  |
| Length range (bp) | 373-464 | 1137-1172 | 483-524 | 891-929 | 859-873 | 393-428 | 982-1014 | 802-956 |  | 849-875 | 928-1000 | 846-847 |  |
| Aligned length (bp) | 475 | 1239 | 546 | 955 | 896 | 432 | 1055 | 961 |  | 913 | 1000 | 847 |  |
| Excluding gaps/missing | 357 | 1096 | 479 | 891 | 845 | 390 | 942 | 755 |  | 824 | 928 | 846 |  |
| Sites with alignment gaps | 117 | 143 | 67 | 64 | 51 | 42 | 113 | 206 |  | 89 | 72 | 1 |  |
| Nucleotide diversity and polymorphism |  |  |  |  |  |  |  |  |  |  |  |  |  |
| Conserved sites | 334 | 1048 | 461 | 850 | 813 | 372 | 885 | 717 |  | 796 | 881 | 759 |  |
| Variable sites | 25 | 48 | 18 | 41 | 32 | 18 | 57 | 38 |  | 28 | 47 | 38 |  |
| Parsimony informative sites (PI) | 15 | 26 | 9 | 22 | 16 | 8 | 28 | 20 |  | 11 | 36 | 21 |  |
| Singleton sites | 10 | 22 | 9 | 19 | 16 | 10 | 29 | 18 |  | 17 | 11 | 17 |  |
| Nucleotide diversity (π) | 0.0137 | 0.0069 | 0.0070 | 0.0083 | 0.0059 | 0.0072 | 0.1029 | 0.0074 |  | 0.0043 | 0.0077 | 0.0080 |  |
| Number of haplotypes | 18 | 17 | 16 | 20 | 15 | 15 | 22 | 18 |  | 17 | 19 | 18 |  |
| Haplotype diversity | 0.952 | 0.692 | 0.911 | 0.942 | 0.917 | 0.919 | 0.97 | 0.958 |  | 0.917 | 0.962 | 0.951 |  |
| Insertion/Deltions (InDels) |  |  |  |  |  |  |  |  |  |  |  |  |  |
| Number of InDel events | 9 | 25 | 14 | 7 | 15 | 12 | 24 | 37 |  | 23 | 2 | 1 |  |
| Average InDel length event | 33.7 | 6.5 | 8.4 | 9.1 | 5.7 | 8.1 | 6.8 | 8.3 |  | 6.0 | 45.0 | 1.0 |  |
| Average InDel length | 30.1 | 8.5 | 9.7 | 9.3 | 6.0 | 6.7 | 7.8 | 8.7 |  | 8.0 | 70.2 | 1.0 |  |
| Number of InDel haplotypes | 9 | 18 | 14 | 7 | 17 | 11 | 24 | 19 |  | 22 | 3 | 2 |  |
| InDel haplotype diversity | 0.625 | 4.192 | 0.923 | 0.558 | 0.946 | 0.78 | 0.962 | 0.954 |  | 0.972 | 0.179 | 0.063 |  |
| InDel diversity | 1.169 | 4.192 | 3.254 | 0.714 | 3.135 | 1.95 | 0.000 | 6.988 |  | 5.520 | 0.238 | 0.063 |  |
| Number of informative InDels^4^ | 7 | 15 | 6 | 5 | 7 | 5 | 8 | 15 |  | 6 | 2 | 0 |  |
| Parsimony informative InDels^5^ | 1 | 8 | 6 | 1 | 4 | 4 | 5 | 12 |  | 4 | 1 | 0 |  |
| Informative singleton InDels | 6 | 7 | 0 | 4 | 3 | 1 | 3 | 3 |  | 2 | 1 | 0 |  |
| PIC (%)^6^ | 8.96 | 5.08 | 4.4 | 4.82 | 4.35 | 5.32 | 6.16 | 5.52 |  | 3.72 | 5.28 | 4.49 |  |

^1^Only the spacer sequences were included for the intergenic regions.

^2^Intron sequence of *rps16* gene and ^3^exon sequences of *accD* or *ycf1b* within the primers were included.

^4^Gaps of >2 bp, not in mononucleotide repeat regions, were considered.

^5^Parsimony informative InDels: InDels that appear in more than two taxa.

^6^Potentially Informative Characters (PIC): (Informative InDels + substitution)/aligned length (%).

**Table S6.** Likelihood ratio tests to identify positively selected sites within the *accD* and *ycf1* coding sequences across the 32 *Artemisia* plastomes

| Gene | Model compared | Df^1^ | -2d*ln*L^2^ | LRT^3^ *p*-value | Positive sites^4^ |
| --- | --- | --- | --- | --- | --- |
| *accD* | M1a (neutral) vs. M2a (selection) | 2 | 37.629838 | 0.000000007 |  |
|  | M7 (beta) vs. M8 (beta & ω >1) | 2 | 33.761316 | 0.000000047 | 211 R 0.998**, 213 A 0.971*,  214 I 0.997**, 215 R 0.997**,  229 V 0.965*, 297 E 1.000**,  314 R 0.955*, 462 A 0.971* |
|  | M8a (ω=1) vs. M8 (selection) | 1 | 31.008058 | 0.000000026 |  |
| *ycf1* | M1a (neutral) vs. M2a (selection) | 2 | 44.953558 | 0.000000000 |  |
|  | M7 (beta) vs. M8 (beta & w >1) | 2 | 45.797814 | 0.000000000 | 623 I 0.997**, 770 K 0.972*,  850 F 1.000**, 1012 R 0.977*, 1059 F 0.975*, 1418 L 0.984*, 1523 K 1.000**, 1604 F 0.988* |
|  | M8a (ω=1) vs. M8 (selection) | 1 | 44.952892 | 0.000000000 |  |

^1^Degree of freedom.

^2^Difference between the log likelihood values.

^3^LRT, Likelihood Ratio Test.

^4^Sites of potentially under positive selection were suggested by the Bayes Empirical Bayes values higher than 0.95 and marked with asterisks (*, > 0.95; **, > 0.99).

**Table S7.** Polymorphic LSRs identified in the 32 *Artemisia* plastomes

| **Structural region** | **Repeat type^1^** | **Unit sequence (5’ to 3’)** | **Minor allele^2^** | **Region** | **Locus** |
| --- | --- | --- | --- | --- | --- |
| LSC | d (×4) | AAAGTAATAAATATATG (17 bp) | gme, ann, fuk, nak | IGS | *trnH-psbA* |
|  | d (×2) | TTTTATTTATGAAATC (16 bp) | pri | IGS | *trnH-psbA* |
|  | d (×2) | TTAGATTATTTAGT (14 bp) | ann, fuk, nak | intron | *trnK-UUU* |
|  | p | AAATAGATCTATTT (14 bp) | kei | CDS | *matK* |
|  | d (×2) (40bp spacer) | AAAAGGATTCTAT (13 bp) | pri, arg, argyro | IGS | *trnK-UUU_rps16* |
|  | d (×2) (40bp spacer) | ATTCTATCAAAAA (13 bp) | pri, arg, argyro | IGS | *trnK-UUU_rps16* |
|  | d (×2) | AATAAAAAGAAATATCATTGTTAAATA (27 bp) | fre, api | IGS | *rps16_trnQ-UUG* |
|  | p | TTAAATATTTAA (12 bp) | fre | IGS | *rps16_trnQ-UUG* |
|  | d (×2) | AACGAATTCCCTTTATTTA (19 bp) | fri (segregating), jap, cap | IGS | *trnS-GCU_trnC-GCA* |
|  | d (×2) (6bp spacer) | TATAATATAAA (11 bp) | jap, cap, kei, api, ann, fuk, nak, | IGS | *trnS-GCU_trnC-GCA* |
|  | d (×2) | CAAGACTTCCAG (12 bp) | mon | IGS | *trnC-GCA_petN* |
|  | d (×2) | TTTCAGATTCATTGGAA (17 bp) | sel | IGS | *petN_psbM* |
|  | d (×2) | TAGGAAGGAAGCTTAATC (18 bp) | hal | CDS/IGS | *rpoB_rpoC1* |
|  | d (×2) | TTTATTATCTTTTATTTG (18 bp) | fri (segregating) | IGS | *trnR-UCU_trnG-UCC* |
|  | d (×2) | TATTAATTAGTGTCTATT (18 bp) | fri (segregating) | IGS | *trnR-UCU_trnG-UCC* |
|  | d (×3) | TAATATGATAG (11 bp) | sel | IGS | *trnR-UCU_trnG-UCC* |
|  | d (×2) | TATATAAGAGAGGGTATAGAG (21 bp) | sie | IGS | *trnR-UCU_trnG-UCC* |
|  | d (×2) | TTTTTAGTTACATA (14 bp) | kei, api | IGS | *trnG-UCC_trnT-GGU* |
|  | d (×2) | TTTATATCTATA (12 bp) | gme (segregating) | IGS | *trnT-GGU_psbD* |
|  | d (×2) | TCTATAGATATT (12 bp) | fre, gme, api | IGS | *trnT-GGU_psbD* |
|  | d (×2) | TTTACGAATAATGTGGTAT (19 bp) | fre, sel, hal | IGS | *trnS-UGA_lhbA* |
|  | d (×2) | TCTTTAGAGATATACCTTTTTAT (23 bp) | gme | IGS | *trnF-GAA_ndhJ* |
|  | d (×3) | AATACATACAA (11 bp) | ann, fuk, nak | IGS | *trnF-GAA_ndhJ* |
|  | d (×2) | TTCTATATTAGTT (13 bp) | jap | IGS | *ndhC_trnV-UAC* |
|  | d (×2) | TTTATTTATTTATATATTATATTTAAATTTTTT (33 bp) | fuk, nak | IGS | *ndhC_trnV-UAC* |
|  | p | TTTGAATATTA (11 bp) | jap, cap, api | IGS | *ndhC_trnV-UAC* |
|  | h (22 bp loop) | ACTTATTAGAT (11 bp) | jap, cap | IGS | *trnM-CAU_atpE* |
|  | d (×2) (13 bp spacer) | AAAAGAGATAA (11 bp) | kei, fuk, nak | IGS | *rbcL_accD* |
|  | d (×2) (10 bp spacer) | GTTCGTTCTCTTAA (14 bp) | kei, fuk, nak | IGS | *rbcL_accD* |
|  | d (×5) | ACTAGAAAGTTCTACCGA (18 bp) | sel | CDS | *accD* |
|  | d (×2) | TAATATATAAAACTAAATAAA (21 bp) | sel | IGS | *accD_psaI* |
|  | d (×2) | ATTTAGAATTAGAATAGATT (20 bp) | pri, arg, argyro, mon, sto | IGS | *accD_psaI* |
|  | d (×2) | GGGGAAGAAAGGG (13 bp) | jap | IGS | *trnP-UGG_psaJ* |
|  | d (×2) | ATTATCCTTGTCTTTGTTTATG (22 bp) | gme, fre, sie | IGS/CDS | *rpl36* |
|  | d (×2) | ATATCTTATTTGTAT (15 bp) | mon | IGS | *rpl14_rps3* |
|  | d (×2) | TTTCTTATATTTATCTA (17 bp) | api | IGS | *rpl14_rps3* |
|  | d (×2) | TTGATGCTTTATTACACA (18 bp) | sie | IGS | *rpl14_rps3* |
| IR | d (×2) | GTTGTGTTGAAGGGATATCTA (21 bp) | fri (segregating) | IGS | *rps12_ycf15* |
|  | d (×2) | TAATTCATATTAATTCATATTA (22 bp) | sie | IGS | *rps7-ycf15* |
|  | d (×2) | TCTTTCTATTTCTTTTCTATATATGG (26 bp) | fre | IGS | *trnN-GUU_ycf1p* |
| SSC | p | AATTTAATTAATTAAATT (18 bp) | sie | IGS | *ψycf1_ndhF* |
|  | d (×3) | ATAATAGGAAAGTAGGAAAATA (22 bp) | kei, dra, sie, fri, gme, | IGS | *ndhF_rpl32* |
|  | d (×2) | ATAAGATAAGAAATAATAA (19 bp) | fuk, nak, gme, sie, sel | IGS | *ndhF_rpl32* |
|  | P | TTAATATTAAA (11 bp) | sie, sel, ann, fuk, nak | IGS | *rpl32_trnL-UAG* |
|  | d (×2) | ATATACTAAAAA (12 bp) | fri (segregating) | IGS | *rpl32_trnL-UAG* |
|  | d (×2) | CAATGAATAATCAAT (15 bp) | ann, fuk, nak, pri, arg, argyro, rub, mon, sto, fed | IGS | *rpl32_trnL-UAG* |
|  | d (×2) | TTATTTAAAATTTAAGAAAATAA (23 bp) | sie | IGS | *ccsA_ndhD* |
|  | d (×2) | CGAATAATGAATAAAAAA (18 bp) | sel | intron | *ndhA* |
|  | d (×2) | TTATTTAAAATTTAAGAAAATAA (23 bp) | sie | IGS | *ccsA_ndhD* |
|  | d (×2) | ATCAATATAAT (11 bp) | mixed | IGS | *ndhG-ndhI* |
|  | d (×2) | CGCCTCTTGTTTTCCTTTGATATTTGGATTTTCACT (36 bp) | sel | CDS | *ycf1* |
|  | d (×2) | TTTTGATTGGA (11 bp) | Sect. *Dracunculus* (all species) | CDS | *ycf1* |

^1^Repeat types are direct tandem repeat (d), hairpin with or without loop spacer (h), or palindromic (p). (×2), duplicated.

^2^Minor alleles identified from specific taxa are presented. Taxa names are presented as the first three letters of specific names given in Table 1.

**Table S8.** *In silico* PCR analysis of the accD-Asteraceae and ycf1b-Asteraceae markers

| Alignment option | Barcode primer | Stringency criteria | |
| --- | --- | --- | --- |
|  |  | Weak | Strong |
| Initial searching word size (bp) |  | 4 | 11 |
| Important size of 3’-end with mismatches (bp) | | 15 | 16 |
| Mismatches allowed in last 15 bases of 3’-end (bp) | | 3 | 1 |
| Minimal complement primer Length (bp) |  | 18 | 18 |
| Local alignment similarity (%) |  | 70 | 85 |
| PCR success^*^ | accD-Asteraceae-F/R | 193/193 | 193/193 |
|  | ycf1b-Asteraceae-F/R | 178/178 | 171/178 |

^*^Simulated by the *in silico* PCR procedure in FastPCR program (v6.0) with two criteria.
